# Reaping the Potential of Wild *Cajanus* Species through Pre-Breeding for Improving Resistance to Pod Borer, *Helicoverpa armigera,* in Cultivated Pigeonpea (*Cajanus cajan* (L.) Millsp.)

**DOI:** 10.3390/biology11040485

**Published:** 2022-03-22

**Authors:** Shivali Sharma, Jagdish Jaba, Polneni Jaganmohan Rao, Suraj Prasad, Nammi Tripura Venkata Venu Gopal, Hari Chand Sharma, Benjamin Kilian

**Affiliations:** 1 International Crops Research Institute for the Semi-Arid Tropics (ICRISAT), Patancheru 502324, Telangana, India; j.jagdish@cgiar.org (J.J.); s.suraj@cgiar.org (S.P.); venu2609@gmail.com (N.T.V.V.G.); hcsh19@gmail.com (H.C.S.); 2 Global Crop Diversity Trust, 53113 Bonn, Germany; benjamin.kilian@croptrust.org; 3 Regional Agricultural Research Station, Professor Jayashankar Telangana State Agricultural University, Warangal 500030, Telangana, India; polneni_rao@yahoo.co.in

**Keywords:** wild *Cajanus*, pigeonpea, pod borer, *Helicoverpa*, biotic stresses, pre-breeding, biochemical compounds, trichomes

## Abstract

**Simple Summary:**

Pigeonpea is an important legume crop that is severely affected by various insect pests, especially pod borer. Because there are low resistance levels to pod borers in the cultivated pigeonpea gene pool, it is necessary to introduce resistance-related traits from its wild relatives. In this study, we conducted a series of crosses to introduce traits related to pod borer resistance from two wild relatives of pigeonpea into two popular cultivated varieties. We generated populations from four different crosses and screened these populations for traits related to pod borer resistance: i.e., low levels of insect damage, high concentrations of insect-deterring compounds in the seeds, and the presence of trichomes on the leaves. The most promising lines were tested across seasons and locations. Ultimately, we identified 21 lines with excellent traits related to pod borer resistance. These lines will be useful for breeding new insect-resistant pigeonpea cultivars. The availability of such cultivars will reduce the use of pesticides to control pests on pigeonpea crop.

**Abstract:**

Pod borer (*Helicoverpa armigera*) causes the highest yield losses in pigeonpea, followed by pod fly (*Melanagromyza obtusa*). High levels of resistance to pod borer are not available in the cultivated genepool. Several accessions of wild *Cajanus* species with strong resistance, and different resistance mechanisms (antixenosis and antibiosis) to pod borer have been identified. These accessions can be utilized to improve the pod borer resistance of cultivated pigeonpea. Using pod borer resistant *Cajanus* *scarabaeoides* and *Cajanus acutifolius* as pollen donors and popular pigeonpea varieties as recipients, pre-breeding populations were developed following simple- and complex-cross approaches. Preliminary evaluation of four backcross populations consisting of >2300 introgression lines (ILs) under un-sprayed field conditions resulted in identifying 156 ILs with low visual damage rating scores (5.0–6.0) and low pod borer damage (<50%). Precise re-screening of these ILs over different locations and years resulted in the identification of 21 ILs having improved resistance to pod borer. Because these ILs were derived from wild *Cajanus* species, they may contain different alleles for different resistance components to pod borer. Hence, these ILs are ready-to-use novel and diverse sources of pod borer resistance that can be utilized for improving the pod borer resistance of cultivated pigeonpea.

## 1. Introduction

Pigeonpea (*Cajanus cajan* (L.) Millsp.) is an often-cross-pollinated diploid (2n = 2x = 22) grain legume crop that is cultivated across an area of about 6.09 m ha with 5.01 m t production and 0.8 t ha^−1^ productivity in the tropical and subtropical regions of Asia and Africa [1]. Although this crop has multiple uses, it is primarily cultivated for its protein-rich seeds. India is the largest producer of pigeonpea, accounting for more than 93% of global production. It is commonly known as arhar, red gram, or tur in India, and is considered as the second most important pulse crop after chickpea. Insect pests continue to be the major biotic constraints to grain legume production, especially the pod borer complex, which causes estimated annual losses of more than USD 2 billion in the semi-arid tropics, despite the application of insecticides costing at least USD 500 million annually [2]. The most damaging pest to pigeonpea crops worldwide is pod borer, *Helicoverpa armigera* (Hübner), which causes the maximum yield losses (25–70%), followed by pod fly, *Melanagromyza obtusa* Malloch (10% losses), spotted borer, *Maruca vitrata* Fabricius (5–25% losses), and pod-sucking bug, *Clavigralla gibbosa* Spinola (10–30% losses) [3,4]. The frequent occurrence of pod borer often results in complete crop failure. The economic losses due to biotic factors have been estimated to be USD 8.48 billion. Pod borer alone may cause losses of more than USD 300 million annually, whereas yield losses caused by pod fly have been estimated at USD 256 million annually [5]. A wide range of insecticides are used to control pod borer under field conditions, but the indiscriminate use of pesticides has led to pesticide resistance, resurgence of pests, and secondary outbreaks of minor pests. The development of resistant cultivars by exploiting host plant resistance is the most effective and eco-friendly solution for the sustainable management of insect pests, including pod borers. Unfortunately, high levels of resistance to pod borer are not available in the cultivated genepool. Therefore, it is necessary to exploit new and diverse sources of resistance.

Crop wild relatives, especially *Cajanus scarabaeoides, Cajanus acutifolius,* and *Cajanus platycarpus* have been identified as potential sources of resistance to pod borer [6,7,8,9]. Different biochemical and morphological mechanisms conferring resistance to the pod borer complex, including antixenosis (oviposition non-preference by insects), antibiosis, and trichomes, have been identified in these wild *Cajanus* species. Therefore, they represent potential sources of resistance genes for introgression into the cultigens. The present investigation was carried out to exploit the potential of two wild *Cajanus* species, *C. acutifolius,* and *C. scarabaeoides,* to improve resistance to pod borer, *H. armigera* in the popular pigeonpea cultivars, ICPL 87119 (Asha) and ICP 8863 (Maruti). We used simple and complex cross approaches to generate introgression lines (ILs), and then identified those with high levels of resistance to the pod borer for further use in pigeonpea breeding programs.

## 2. Materials and Methods

### 2.1. Population Development

The pollen donors were *C. acutifolius* accession ICPW 001, which has high levels of antixenosis for ovipositing insects and antibiosis [8], and the *C. scarabaeoides* accession ICPW 281, which has a high density of C-type trichomes. The recipients were the two popular pigeonpea varieties ICPL 87119 and ICP 8863. Using these donors and recipients, four pre-breeding populations were developed following simple and complex cross approaches at the International Crops Research Institute for the Semi-Arid Tropics (ICRISAT), Patancheru, India. ICPL 87119, popularly known as ‘Asha’, is a high-yielding, medium-duration leading variety widely cultivated in India [10]. ICP 8863, also known as ‘Maruti’ [11], is a medium-duration high-yielding pigeonpea variety resistant to *Fusarium* wilt.

**Figure 1:**
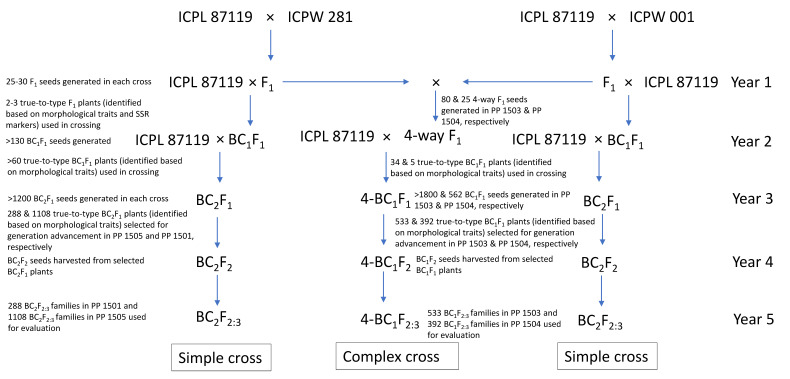
Breeding scheme for generating pre-breeding populations following simple and complex cross approach in pigeonpea.

The breeding scheme used to generate the pre-breeding populations is given in Figure 1. The pigeonpea varieties ICPL 87119 and ICP 8863 were used as the female parent, and the wild species accessions were used as the pollen parent to generate F_1_ hybrids. In each cross, true F_1_s were identified based on the morphological traits such as growth habit, leaf shape, stem color, flower color and streak pattern, days to first flowering, and pod traits, as well as by analysis of highly polymorphic simple sequence repeat (SSR) markers. For testing the hybridity of F_1_ plants, DNA was extracted from the leaves collected after one month of germination and five highly polymorphic SSR markers (CcM0724, CcM0402, CcM0047, CcM0306, and CcM0494) were tested on each cross combination. All the true F_1_ plants derived from the cross-involving *C. acutifolius* (ICP 8863 × ICPW 001) were semi-fertile (30–40% pollen fertility) to completely sterile (10–20% pollen fertility). In this cross, true F_1_ plants were used as female parents and subsequently backcrossed with ICP 8863 to produce BC_1_F_1_ seeds. In contrast, all the F_1_ plants derived from the cross-involving *C. scarabaeoides* (ICPL 87119 × ICPW 281) were highly fertile (>80% pollen fertility) and were used as the pollen parent with ICPL 87119 to produce BC_1_F_1_ seeds. This was performed to ensure identification of true BC_1_F_1_ crosses from selfed F_2_s. The true BC_1_F_1_ plants in both crosses were identified on the basis of morphological traits. The confirmed BC_1_F_1_ plants were used for the second backcross with the respective cultivated parent to produce BC_2_F_1_ seeds. True BC_2_F_1_ plants were selfed twice to produce BC_2_F_3_ populations in both crosses. The pre-breeding population derived from the cross ICP 8863 × ICPW 001 consisting of 1108 lines was designated as PP 1501; and that derived from the cross ICPL 87119 × ICPW 281 consisting of 288 lines was designated as PP 1505 (Table 1; Figure 1).

With a view to combining different components governing pod borer resistance into a common genetic background, we also developed two backcross populations (four-way BC_1_F_2_) derived from complex four-way F_1_ crosses in two different genetic backgrounds, ICPL 87119 ((ICPL 87119 × ICPW 1) × (ICPL 87119 × ICPW 281)), and ICP 8863 ((ICP 8863 × ICPW 1) × (ICP 8863 × ICPW 281)). To generate a complex cross population in the ICPL 87119 background, sterile and semi-sterile F_1_ plants from the ICPL 87119 × ICPW 1 cross were crossed with fertile F_1_ plants from the ICPL 87119 × ICPW 281 cross. These four-way F_1_ plants were further crossed with ICPL 87119 to generate BC_1_F_1_ seeds, designated as 4-BC_1_F_1_. A similar approach was followed to generate 4-BC_1_F_1_ seeds in the ICP 8863 background. True 4-BC_1_F_1_ plants were selfed twice to produce 4-BC_1_F_3_ populations from both crosses. The pre-breeding population derived from the cross ((ICPL 87119 × ICPW 1) × (ICPL 87119 × ICPW 281)) consisting of 533 ILs was designated as PP 1503; and that derived from the cross ((ICP 8863 × ICPW 1) × (ICP 8863 × ICPW 281)) consisting of 392 ILs was designated as PP 1504 (Table 1; Figure 1).

### 2.2. Evaluation of Pre-Breeding Populations for Pod Borer Complex

Four backcross pre-breeding populations, PP 1501, PP 1505, PP 1503, and PP 1504 consisting of 1108, 288, 533, and 392 ILs, respectively, were evaluated for pod borer damage under un-sprayed field conditions during the 2018 rainy season. In each population, the ILs along with susceptible (ICPL 87, ICPL 85010), moderately susceptible (ICPL 88039, ICP 7035) and moderately resistant checks (ICPL 87119, ICPL 332WR, and ENT 11) were planted in a single row plot (4 m long). At least one check was planted after every six ILs in each single row.

Visual damage to pods was scored using a rating on a scale of 1 to 9 (1.0: almost no damage, resistant; 9.0: severely damaged, highly susceptible) at the podding stage. The recovery resistance score was recorded at harvest stage on a scale of 1 to 9 (1.0: plants with <10% pod damage, showing good recovery from insect damage in the first flush, and the pod uniformly distributed throughout the plant; 9.0: plants with >80% pod damage, very poor recovery from insect damage, and <20% of the pods retained on the plant) [12]. A total of 156 ILs with recovery resistance scores of 5.0 to 6.0 were selected and their pod damage was estimated. In each of the 156 lines, 100 pods were randomly selected, and each pod was critically examined for damage caused by pod borer, pod fly, plume moth (*Exelastis atmosa* Wals.), and pod wasp (*Tanaostigmodes cajaninae* La Salle).

The selected ILs were re-evaluated using a randomized block design (RBD) in the black soil (Vertisols) precision fields under un-protected field conditions during the 2019 rainy season at Patancheru (17°51′ N, 78°27′ E; 545 m) and at Warangal (18°00′ N, 79°59′ E; 262 m) locations. Depending upon seed availability, 156 ILs at Patancheru and 136 ILs at Warangal along with susceptible (ICPL 87, ICPL 85010), moderately susceptible (ICPL 88039, and ICP 7035), and moderately resistant checks (ICPL 87119, ICPL 332WR, and ENT 11) were randomized and grown in three replications during the 2019 rainy season. The seeds were sown in triplicate on ridges 75 cm apart. There were four rows in each plot, and each row was 4 m long. The plants were thinned to 30 cm spacing between plants at 30 days after seedling emergence. Standard agronomic practices were followed for raising the crop, including application of basal fertilizer (nitrogen: phosphorus: potassium 100:60:40 kg ha^−1^) and top dressing (urea 50 ha^−1^). A fungicide (metalaxyl) spray (1.0 kg active ingredient (ai) ha^−1^) was applied to control *Fusarium* wilt. The same set of lines was evaluated for pod borer resistance by conducting pod assessment, pod bioassays, and analyses of biochemical traits under laboratory conditions. To confirm the results, the most promising 39 pod borer-tolerant lines were selected for re-evaluation during the 2020 rainy season following the same procedure. The pest susceptibility (%) of each line was calculated based on pod borer damage and pod borer complex damage in 39 lines over years using the formula derived from Abbott (1925) [13].

Pest susceptibility (%) = ((Pod damage in susceptible check − Pod damage in test entry)/Pod damage in susceptible check) × 100

The pest susceptibility (%) was then converted to a 1 to 9 pest susceptibility rating (PSR) adopting the following scale [14]:
**Pest Susceptibility (%)****Pest Susceptibility Rating (PSR)****Category**100%1Highly resistant75%2Resistant50% to 75%3Resistant25% to 50%4Moderately resistant10% to 25%5Moderately resistant−10% to 10%6Moderately susceptible−25% to −10%7Susceptible−50% to −25%8Susceptible−50% or less9Highly susceptible

### 2.3. Criteria for Examination of Pod Borer Damage

Each pod was carefully examined for any damage caused by the main pests, as follows:

1. Healthy or clear pods having no external damage symptoms;
2. Pods damaged by pod borer, *H. armigera* (big circular holes without larval exuviae on the pods);
3. Pods damaged by pod fly (minute holes on pods);
4. Pods damaged by pod wasp (minute holes at upper side of pod tip, empty pods, and pod length drastically shortened);
5. Pods damaged by plume moth (two to three medium-sized circular holes on pods);
6. The *Maruca* (spotted pod borer) damaged pods have small, darkened entry holes with frass-fecal matter and chewed remains of the pods around the entry holes. It also has a typical symptom with holes in pods at one end [15].

The numbers of healthy and damaged pods due to pod borer complex (pod borer, pod fly, pod wasp, and plume moth) were recorded and converted into percentage pod damage, as follows: % pod damage = ((number of damaged pods)/(total number of pods)) × 100.

### 2.4. Procedure for Recording the Larval Count

At podding stage, three plants were randomly selected, and tagged with distinct color labels in all the three replications. The observation was recorded on the number of *H. armigera* larvae at podding stage on each tagged plant in each replication.

### 2.5. Helicoverpa Armigera Culture

The neonates of *H. armigera* used in bioassays were obtained from a laboratory-reared culture at ICRISAT, Patancheru, India. The *H. armigera* larvae were reared individually in the laboratory on a chickpea-based artificial diet [16] under the following conditions: 27 ± 2 °C, 65% to 75% relative humidity, and a 16 h:8 h (L/D) photoperiod.

### 2.6. Detached Pod Assay to Assess Antibiosis Mechanism of Resistance in Pigeonpea ILs Using Third-Instar Larvae of Pod Borer, H. armigera

The relative resistance of pigeonpea ILs was evaluated using third-instar larvae of *H. armigera*. Detached inflorescences with pods were cut with a surgical blade and immediately placed in a slanting direction onto 3% *w/v* agar–agar medium in a 250 mL plastic cup (9.0 × 6.5 cm diameter) [2]. There were three replications of each accession in an RBD. A single third-instar larva was released on pods of pigeonpea with two pods per plastic cup. The initial and final larval weights were recorded before and after a 4-day-feeding period, respectively, and the pod damage rating was determined at the end of the feeding period. The weight gained (in percentage) by the larvae was calculated as follows: weight gain (%) = ((final larval weight − initial larval weight)/initial larval weight) × 100.

### 2.7. Biochemical Profiling of Seeds

Biochemical parameters, i.e., total phenols and total flavonoids concentrations, were determined for the seeds of 156 ILs as well as those of resistant and susceptible checks. To determine total flavonoids and phenols concentrations, the seeds of each accession were oven-dried at 55 °C for 3 days, then powdered in a Willey mill (Thomas Willey Mills, Swedesboro, NJ, USA) and defatted using hexane solution (100 mL g^−1^). The resulting materials were used for analyses of total phenols and total flavonoid concentrations using spectrophotometric methods, as described below. Each line was analyzed with two replicates in a completely randomized design [4].

#### 2.7.1. Estimation of Total Phenols

The total phenols concentration in seeds was determined using a colorimetric method [17]. A 0.5 g portion of defatted seed sample was ground with 80% (*v*/*v*) ethanol in a pestle and mortar and then the mixture was centrifuged at 10,000 rpm for 20 min. The extraction was repeated five times. The supernatant was evaporated to dryness and then dissolved in water (5 mL). The total phenols concentration was expressed in mg/g of dry weight of seeds.

#### 2.7.2. Estimation of Total Flavonoids

The total flavonoids concentration in seeds was determined by vanillin reagent method [18]. A 0.5 g portion of defatted seed sample was homogenized in ethanol and the mixture was centrifuged at 10,000 rpm for 20 min. The supernatant was evaporated to dryness and then dissolved in water (5 mL). This mixture was used for estimation of the total flavonoid concentration (expressed in mg/g of dry weight of seeds).

### 2.8. Morphological Parameters

**Figure 2:**
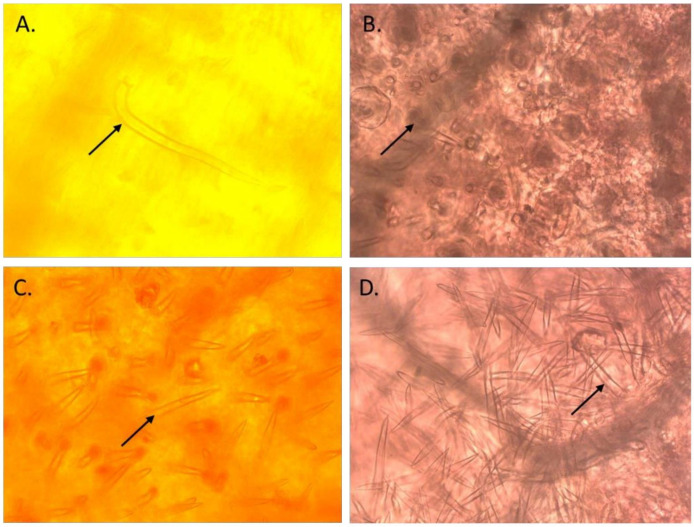
Different types of trichomes observed in the introgression lines of pigeonpea derived from wild *Cajanus* species. (**A**): Type A trichomes; (**B**): Type B trichomes; (**C**): Type C trichomes; and (**D**): Type D trichomes.

On the basis of the performance of selected ILs during the 2019 rainy season, the most promising 39 pod borer resistant ILs were selected for further analysis in a trial in the 2020 rainy season. The trichome density on the leaves of these 39 ILs was recorded by observing a minimum of three uniformly developed leaves from each accession. Each IL was analyzed with three replications. The leaf samples were immersed in an acetic acid and ethanol mixture (2:1) in stoppered 10 mL glass vials for 24 h to remove chlorophyll, and subsequently transferred into lactic acid (90% *v*/*v*) as a preservative. The calyxes and the pods were examined at 10× magnification under a stereomicroscope (Carl Zeiss, Inc., Thornwood, NY, USA) equipped with an ocular measuring grid. The number of different types of trichomes (types A, B, C, and D; Figure 2) and their density (mm^2^) within the microscopic field were recorded [4,7].

### 2.9. Statistical Analysis

Data were subjected to analysis of variance (ANOVA) using GenStat software (14th Edition). The significance of differences between the genotypes was determined by F-test, while differences among treatment means were determined by the least significant difference (LSD) test at *p* ≤ 0.05. The mean performance of the test entries was assessed across seasons.

## 3. Results

### 3.1. Field Evaluations of Pod Borer Complex Damage in Four Populations during the 2018 Rainy Season

#### 3.1.1. PP 1501

In PP 1501, the visual damage rating at the podding stage ranged from 6 to 9 and the recovery resistance score at the harvest stage ranged from 4 to 7. Total 243 ILs with recovery resistance scores of 4 to 6 along with the resistant and susceptible checks were selected to evaluate damage caused by the pod borer complex.

The pod borer (*H. armigera*) damage ranged from 7% to 48% (Table 2). The lines PP1501-14-4-3, PP1501-16-7-2, and PP1501-16-7-6 exhibited the lowest pod borer damage (7%) followed by PP1501-1-12-3 (9%); PP1501-1-2-2 and PP1501-2-5-1 (10%); PP1501-1-8-2 and PP1501-2-3-6 (12%); and PP1501-1-22-1, PP1501-2-2-5, PP1501-2-13-2, and PP1501-14-1-5 (all 14%) (Supplementary Table S1). The pod borer damage was lower in the cultivated recurrent parent ICP 8863 (18%) than in the checks ENT 11 (29%), ICPL 332WR and ICPL 87119 (20%) and ICPL 87 (33%) (Table 2).

The pod fly (*M. obtusa*) damage ranged from 3% to 55%. The lowest pod fly damage was recorded in PP1501-9-18-1 (3%) followed by PP1501-1-13-5, PP1501-1-23-2 (10%), PP1501-1-22-5 (12%), PP1501-2-6-2, PP1501-10-17-1, and PP1501-10-17-2 (13%), and PP1501-1-5-2, PP1501-2-16-4, PP1501-4-21-6 and PP1501-12-1-2 (all 14%). The level of pod fly damage varied among the cultivated recurrent parent ICP 8863 (38%) and the checks ENT 11 (30%), ICPL 332WR (34%), ICPL 87119 (40%), and ICPL 87 (44%). Apart from pod borer and pod fly, pod wasp and plume moth also caused a small amount of damage (Table 2, Supplementary Table S1).

The total pod borer complex damage ranged from 33% to 85%. The lowest pod borer complex damage was observed in PP1501-16-7-6 (33%) followed by PP150/1-1-13-5, PP1501-9-18-1 (35%), PP1501-16-7-2 (38%), PP1501-14-7-7 (39%), and PP1501-2-3-6 and PP1501-2-6-2 (41%). The total pod borer complex damage was relatively high in the cultivated recurrent parent ICP 8863 (65%) and the checks ENT 11 (71%), ICPL 332WR (62%), ICPL 87119 (70%), and ICPL 87 (75%) (Table 2, Supplementary Table S1).

From this population, we selected the 79 ILs with the lowest levels of pod borer complex/pod borer/pod fly damage (Table 2) for further field and laboratory evaluations during the 2019 rainy season.

#### 3.1.2. PP 1503

The visual damage rating at the podding stage ranged from 4 to 9 and the recovery resistance score at the harvest stage ranged from 5 to 8. Total 342 lines with recovery resistance scores of 5 to 6 along with the susceptible and resistant checks were further evaluated for pod borer complex damage.

In PP 1503, the pod borer damage ranged from 6% to 40%. The IL PP1503-11-1-1 showed the lowest pod borer damage (6%) followed by PP1503-3-1-3 (11%), PP1503-5-2-4, (14%), PP1503-29-2-1 (15%), and PP1503-18-1-7 (16%) (Supplementary Table S2). The level of pod borer damage ranged from 25% to 30% in the recurrent parent and the checks (30% in the cultivated recurrent parent ICPL 87119 and 25%, 26%, 30%, and 30% in the checks ENT 11, ICPL 332WR, ICPL 87119, and ICPL 87, respectively) (Table 2).

In the same set of ILs, the pod fly damage ranged from 10% to 44%. The lowest pod fly damage was observed in PP1503-29-1-5 (10%) followed by PP1503-22-1-5 (12%), PP1503-5-2-4 (14%), PP1503-6-1-4 (16%), and PP1503-25-5-6 (18%). The level of pod fly damage varied among the cultivated recurrent parent ICPL 87119 (41%) and the checks, ENT 11 (30%), ICPL 332WR (36%), and ICPL 87 (42%). A low incidence of damage by pod wasp and plume moth was recorded (Table 2).

The total pod borer complex damage ranged from 37% to 80%. The lowest pod borer complex damage was recorded in PP1503-25-5-6 (37%) followed by PP1503-5-2-4 and PP1503-6-1-4 (both 39%), PP1503-29-1-5 (40%), PP1503-11-1-4 (41%), and PP1503-18-1-7 (43%). There were high levels of pod borer complex damage in the cultivated recurrent parent ICPL 87119 (75%) and the checks, ENT 11 (60%), ICPL 332WR (67%), and ICPL 87 (77%) (Supplementary Table S2).

On the basis of the lowest pod borer complex damage, we selected 41 ILs (Table 2) for further field and laboratory evaluations during the 2019 rainy season.

#### 3.1.3. PP 1504

The visual damage rating at the podding stage ranged from 6 to 9 and the recovery resistance score at the harvest stage varied from 5 to 7. Ninety-one ILs with recovery resistance scores of 5 to 6 at the harvest stage, along with checks, were further evaluated for pod borer, pod fly, and pod borer complex damage.

The pod borer damage ranged from 9% to 45%. The line PP1504-4-8-2 exhibited the lowest pod damage (9%), followed by PP1504-4-9-1 (11%), PP1504-4-9-2 (13%), and PP1504-2-1-3 (17%) (Supplementary Table S3). The level of pod borer damage varied among the cultivated recurrent parent ICP 8863 (25%) and the checks, ENT 11 (36%), ICPL 332WR (25%), ICPL 87119 (31%), and ICPL 87 (45%) (Table 2).

In the same set of ILs, the pod fly damage ranged from 26% to 50%. The lowest pod fly damage was recorded in PP1504-2-11-5 and PP1504-3-7-2 (both 26%) followed by PP1504-1-3-1 (29%). All of these values were lower than those in the cultivated recurrent parent ICP 8863 (39%) and the checks, ENT 11 (44%), ICPL 332WR (35%), ICPL 87119 (39%), and ICPL 87 (40%). Apart from pod borer and pod fly, there was a low incidence of damage by pod wasp and plume moth (Table 2).

The total pod borer complex damage ranged from 46% to 87% with the lowest level of damage recorded in PP1504-4-9-1 (46%) followed by PP1504-4-8-2 (47%), PP1504-2-11-5 (50%), and PP1504-3-7-2 (51%). The level of pod borer complex damage was relatively high in the cultivated recurrent parent ICP 8863 (67%) and the checks ENT 11 (67%), ICPL 332WR (65%), ICPL 87119 (74%), and ICPL 87 (87%) (Table 2).

On the basis of the lowest levels of pod borer, pod fly, and pod borer complex damage, eight ILs were selected for re-evaluation both under field and laboratory studies during the 2019 rainy season (Table 2).

#### 3.1.4. PP 1505

The visual damage rating at the podding stage ranged from 6 to 9 and the recovery resistance score at the harvest stage ranged from 5 to 8. A total of 393 ILs with recovery resistance scores of 5 to 6 at the harvest stage, along with checks, were further evaluated for pod borer, pod fly, and pod borer complex damage.

The pod borer damage ranged from 5% to 35%. The line PP1505-34-3-6 exhibited the lowest pod damage (5%) followed by PP1505-63-2-4 (7%), PP1505-11-2-4 (8%), PP1505-61-4-1 (9%), PP1505-59-4-4 and PP1505-11-2-5 (10%), PP1505-11-2-6 and PP1505-28-6-1 (11%), and PP1505-2-8-1, PP1505-36-4-4, and PP1505-63-2-6 (12%) (Supplementary Table S4). The level of pod borer damage varied among the cultivated recurrent parent ICPL 87119 (25%) and the checks ENT 11, ICPL 332WR, and ICPL 87 (22%, 21%, and 35%, respectively) (Table 2).

In the same set of ILs, the pod fly damage ranged from 7% to 57%. The lowest pod fly damage was recorded in PP1505-11-2-6 (7%) followed by PP1505-13-6-3 (11%), PP1505-11-2-2 (14%), PP1505-2-3-1, and PP1505-28-6-1 (15%), PP1505-11-2-5 and PP1505-59-7-2 (18%), PP1505-20-5-2 (22%), PP1505-59-4-4, and PP1505-36-4-2 (23%), and PP1505-32-3-2 and PP1505-29-4-1 (24%). The level of pod fly damage varied among the cultivated recurrent parent ICPL 87119 (37%) and the checks ENT 11 (31%), ICPL 332WR (30%), and ICPL 87 (33%) (Supplementary Table S4). There was also a low incidence of damage by pod wasp and plume moth (Table 2).

The total pod borer complex damage ranged from 23% to 80%. The lowest pod borer complex damage was recorded in PP1505-11-2-6 (23%), followed by PP1505-28-6-1 (27%), PP1505-11-2-5 (28%), PP1505-13-6-3 and PP1505-11-2-4 (36%), PP1505-34-3-6 (38%), PP1505-63-2-4 (39%), PP1505-2-3-1 (40%), PP1505-36-4-2 and PP1505-59-4-4 (42%), PP1505-11-2-2 (43%), and PP1505-36-4-1 and PP1505-20-5-2 (44%) (Supplementary Table S4). The pod borer complex damage varied among the cultivated recurrent parent ICPL 87119 (63%) and the checks ENT 11 (58%), ICPL 332WR (54%), and ICPL 87 (80%) (Table 2).

The 28 ILs with the lowest levels of pod borer/pod fly/pod borer were selected for further evaluation in the field and the laboratory during the 2019 rainy season (Table 2).

### 3.2. Evaluation of Selected ILs during the 2019 Rainy Season at Patancheru

#### 3.2.1. Phenotyping under Unprotected Field Conditions

In total, 156 ILs with checks were re-evaluated using a RBD during the 2019 rainy season. We detected significant differences among the ILs for most of the traits (*p* ≤ 0.001; Supplementary Table S5). The visual damage rating at the podding stage ranged from 5 to 7 and the recovery resistance score at the harvest stage ranged from 4.5 to 7.5. Ninety-six ILs with recovery resistance scores of 5 to 6 at the harvest stage, along with checks, were further evaluated to determine the extent of damage by pod borer, pod fly, and pod borer complex and other biochemical traits (Supplementary Table S5).

The pod borer damage ranged from 16% to 34%. The lowest level of damage was recorded in line PP1505-20-5-2 (~17%) and PP1503-11-1-4 (17%), followed by PP1505-32-5-3 (~18%). The highest level of damage was recorded in PP1503-20-1-5 (25%) (Supplementary Table S5). There was a range of damage levels in the cultivated recurrent parents, ICP 8863 (21%) and ICPL 87119 (~22%) and the checks, ENT 11 (18%), ICPL 332WR (18%), and ICPL 87 (34%) (Table 3).

In the same set of ILs, the pod fly damage ranged from 9% to 53%. The lowest pod fly damage was recorded in PP1501-1-22-3 and PP1503-8-2-4 (9%), followed by PP1503-5-3-3 (10%), which was lower than in the cultivated recurrent parents, ICP 8863 (18%) and ICPL 87119 (20%) and the checks, ENT 11 (29%), ICPL 332WR (~22%), and ICPL 87 (35%). The highest pod fly damage was recorded in PP1504-4-9-2 (53%) (*p* ≤ 0.001; Supplementary Table S5). Besides pod borer and pod fly damage, pod wasp and plume moth caused a small amount of damage (Table 3).

The damage caused by the total pod borer complex ranged from 32% to 78%. The lowest pod borer complex damage was recorded in PP1501-2-3-6 (32%) followed by PP1501-1-22-3 and PP1501-3-17-3 (33%), which was lower than in the cultivated recurrent parents, ICP 8863 (42%) and ICPL 87119 (46%), and the checks, ENT 11 (50%), ICPL 332WR (44%), and ICPL 87 (70%) (Table 3). The highest pod borer complex damage was observed in PP1503-29-2-1 (78%) (*p* ≤ 0.001; Supplementary Table S5). 

Besides pod borer complex damage, the number of larvae on each IL at the podding stage in each replication was recorded. The mean larval count per plant ranged from 0.11 to 2.44 (Supplementary Table S5). The lowest larval count was recorded in PP1501-1-10-8, PP1501-12-1-2, PP1501-4-17-7, PP1501-5-14-3, PP1503-16-3-8, PP1503-29-2-7, PP1503-8-2-4, and PP1505-28-6-1 (0.11 larvae per plant) followed by PP1501-16-7-2 and PP1505-63-2-4 (0.22 per plant), and PP1501-2-3-6, PP1501-4-17-3, and PP1505-11-2-6 (0.33 per plant). The highest larval count was recorded in PP1501-4-12-2 and PP1501-4-17-1 (2.0 per plant) (*p* ≤ 0.001; Supplementary Table S5). The mean larval count varied among the cultivated recurrent parents, ICP 8863 (0.33) and ICPL 87119 (0.78) and the checks, ENT 11 (0.89), ICPL 332WR (0.44), and ICPL 87 (2.44 per plant) (Supplementary Table S5).

#### 3.2.2. Estimated Concentrations of Major Biochemical Compounds in Seeds

**Figure 3:**
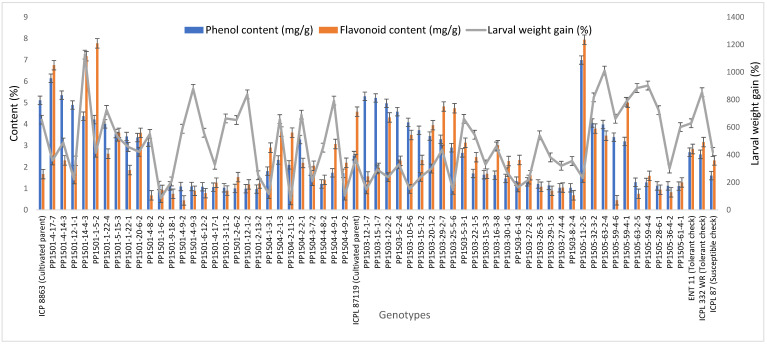
Total phenols and flavonoids concentrations in seeds and larval weight gain in selected ILs, and their cultivated recurrent parents and checks. Evaluations were conducted during the 2019 rainy season at ICRISAT, Patancheru, India. Error bars indicate standard error of the mean.

The concentrations of total phenols and flavonoids differed significantly among the ILs (*p* ≤ 0.001, data not shown). The total phenols concentration in seeds of the ILs and checks ranged from 0.97 to 6.99 mg/g (Figure 3). The highest total phenols content was recorded in PP1505-11-2-5 (6.99 mg/g) followed by PP1501-4-17-7 (6.14 mg/g), PP1501-4-14-3 (5.34 mg/g), PP1503-12-1-7 (5.29 mg/g), PP1503-15-1-7 (5.22 mg/g), PP1503-12-2-2 (4.97 mg/g), PP1501-12-1-1 (4.88 mg/g), PP1503-5-2-4 (4.58 mg/g), PP1501-14-4-3 (4.36 mg/g), PP1501-1-5-2 (4.20 mg/g), PP1503-10-5-6 (4.07 mg/g), and PP1505-32-3-2 (4.04 mg/g) (*p* ≤ 0.001). The total phenols content varied among the cultivated recurrent parents, ICP 8863 (5.11 mg/g) and ICPL 87119 (2.53 mg/g), and the checks ENT 11 (2.69 mg/g), ICPL 332WR (2.59 mg/g), and ICPL 87 (1.60 mg/g) (Figure 3).

The total flavonoids concentration in the seeds ranged from 0.44 to 12.01 mg/g (Figure 3). The highest flavonoids concentration was recorded in PP1503-28-1-3 (12.01 mg/g), followed by PP1503-6-2-5 (10.25 mg/g), PP1503-22-3-1 (10.12 mg/g), PP1505-11-2-5 (7.93 mg/g), PP1501-1-5-2 (7.77 mg/g), PP1501-14-2-1 (7.44 mg/g), PP1501-14-4-3 (7.16 mg/g), PP1501-4-17-7 (6.75 mg/g), and PP1505-11-2-4 (6.07 mg/g) (*p* ≤ 0.001). These values were higher than those in the cultivated recurrent parents, ICP 8863 (1.67 mg/g) and ICPL 87119 (4.58 mg/g), and the checks ENT 11 (2.84 mg/g), ICPL 332WR (3.16 mg/g), and ICPL 87 (2.31 mg/g) (Figure 3).

#### 3.2.3. Detached Pod Bioassay under Laboratory Conditions during 2019 Rainy Season

We detected highly significant differences among the ILs for all traits, viz., pre-larval weight, damage rating, post-larval weight, and % larval weight gain (*p* ≤ 0.001). The pod damage rating, which was recorded at 5 days after the release of larvae on each IL, ranged from 2.33 to 8.50. The lowest pod damage rating was recorded in PP1501-2-13-2, PP1501-4-21-6, and PP1504-1-3-1 (2.33) followed by PP1501-1-6-2, PP1501-1-8-2, PP1501-3-17-3, PP1501-4-8-2, PP1501-4-9-2, and PP1504-2-11-5 (2.67). These values were less than the pod borer damage rating in the tolerant checks, ENT 11 and ICPL 332WR (both 5.67) (Supplementary Table S6). The highest pod damage rating was observed in PP1505-2-3-1 (8.50).

The larval weight gain ranged from 16.8% to 1203.3% (Figure 3). The lowest larval weight gain was recorded in PP1503-3-1-3 (16.8%) followed by PP1501-1-10-8 (45.1%), PP1503-22-4-5 (52.4%), PP1501-4-14-4 (65.5%), PP1504-2-11-5 (69.7%), PP1501-1-6-2 (85.2%), PP1503-15-2-2 (85.9%), PP1501-5-14-1 (88.7%), PP1501-5-14-4 (96.7%), PP1501-12-1-3 (97.8%), and PP1504-4-9-2 (103.4%). These values were less than those of the tolerant checks ENT 11 (628.5%) and ICPL 332WR (854%) (Figure 3, Supplementary Table S6). The highest larval weight gain was recorded in PP1501-1-13-5 (1203.3%) and PP1501-14-4-3 (1126.6%).

On the basis of the re-evaluation of 156 selected ILs for different traits (damage due to pod borer, pod borer complex, damage rating, larval weight gain (%), phenol content, flavonoid content, and larval count at the podding stage), the 39 ILs with the least pod borer damage were selected and evaluated during the 2020 rainy season to confirm their pod borer resistance.

### 3.3. Evaluation of 136 Selected ILs during the 2019 Rainy Season at Warangal, India

#### 3.3.1. Phenotyping for Pod Borer Complex Damage under Unprotected Field Conditions

We re-evaluated the 136 selected ILs, along with checks, using a RBD during the 2019 rainy season. We detected significant differences among the ILs for most of the traits (*p* ≤ 0.001). The recovery resistance score at the harvest stage ranged from 4 to 8. Thirty-five ILs with recovery resistance scores of 4 to 6, along with the checks, were further evaluated to determine pod borer, pod fly, and pod borer complex damage (Supplementary Table S7).

The pod borer damage ranged from 4% to 25%. The lowest pod borer damage was recorded in PP1501-4-17-3 (4%), followed by PP1505-59-4-1 (5%) and PP1503-27-2-8 (6%). The level of pod borer damage in the checks was as follows: ENT 11 (8%), ICPL 332WR (4%), ICPL 87119 (6%), and ICPL 87 (17.6%) (Table 4).

In the same set of ILs, the pod fly damage ranged from 3% to 32%. The lowest pod fly damage was recorded in PP1503-15-1-7 (3%) followed by PP1501-2-16-4 (4%). These values were lower than those in the tolerant checks: ENT 11 (13%), ICPL 332WR (21%), and ICPL 87119 (19%). Besides damage caused by pod borer and pod fly damage, a small amount of damage by pod wasp and plume moth was observed in these ILs (Supplementary Table S7).

The damage caused by pod borer complex varied from 14% to 58%. The lowest pod borer complex damage was recorded in PP1503-15-1-7 (14%) followed by PP1501-2-16-4 (16%) and PP1501-16-7-2 (20%). These values were lower than those in the tolerant checks: ENT 11 (30%), ICPL 332WR (37%), and ICPL 87119 (34%) (Table 4).

#### 3.3.2. Growth Phenotypes of Selected ILs under Unprotected Field Conditions

The ILs took, on an average, about 108 days to first flowering (range, 84–131 days), 116 days to 50% flowering (range, 92–139 days), and 158 days to maturity (range, 135–181 days). These values were similar to those of the cultivated recurrent parents ICP 8863 (97, 105 and 145 days to first flowering, 50% flowering and maturity, respectively), and ICPL 87119 (102, 110, and >161 days to first flowering, 50% flowering, and maturity, respectively) (Table 4).

### 3.4. Confirming Pod Borer Resistance in 39 Selected ILs during the 2020 Rainy Season

**Figure 4:**
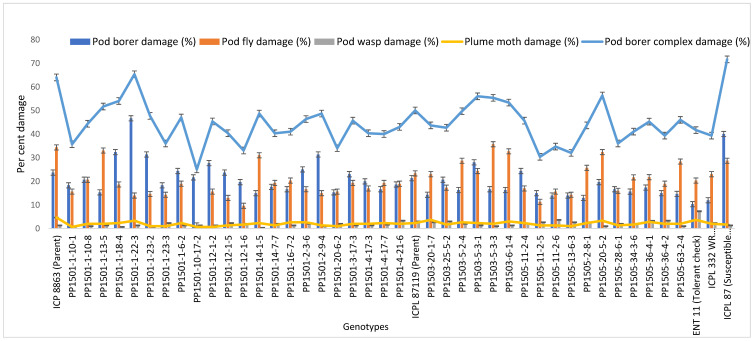
Confirmation of pod borer resistance in the most promising ILs during the 2020 rainy season at ICRISAT, Patancheru, India.

**Figure 5:**
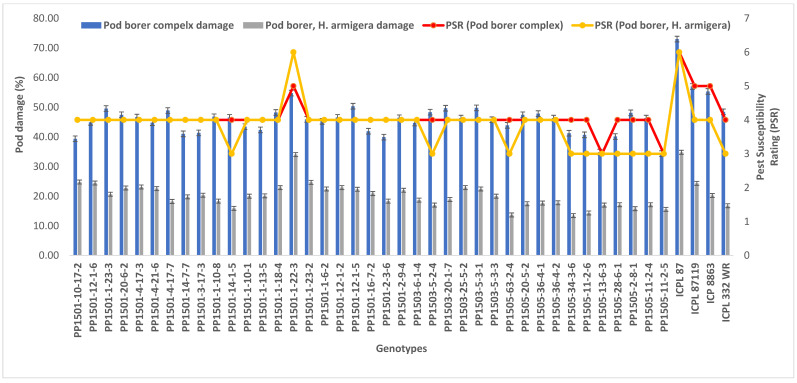
Pest susceptibility rating (PSR) of selected 39 lines based on pod borer complex and pod borer (*H. armigera*) damage over years.

To confirm the above results, the 39 pod borer resistant ILs were re-evaluated using a RBD during the 2020 rainy season. We detected significant differences among the ILs for the recovery resistance score and pod borer complex damage (*p* ≤ 0.05, data not shown). The level of pod borer damage ranged from 13.0% to 46.7%. The lowest pod borer damage was recorded in PP1505-2-8-1 (13%) followed by PP1505-11-2-6 and PP1505-13-6-3 (14%). These values were lower than that of the cultivated recurrent parent ICPL 87119 (over 21%), similar to that of the resistant check ICPL 332WR (12%), and lower than that of the other resistant check ICPL 87 (40%). The pod fly damage ranged from 1.3% to 35.7%. The lowest pod fly damage was recorded in PP1501-10-17-2 (1.3%) followed by PP1501-12-1-6 (9.7%) and PP1505-11-2-5 (11.3%). These values were lower than those of the cultivated recurrent parents ICP 8863 (34.3%) and ICPL 87119 (23.3%), the resistant check ICPL 332WR (23.0%) and the susceptible check ICPL 87 (28.7%). The total pod borer complex damage ranged from 25% to 71.7%. The lowest pod borer complex damage was observed in PP1501-10-17-2 (25%) followed by PP1505-11-2-5 (30.3%) and PP1505-13-6-3 (32%). Thus, the levels of damage in these lines were lower than in the cultivated recurrent parents ICP 8863 (64%) and ICPL 87119 (~50%) and the checks ENT 11 (~42%), ICPL 332WR (39%), and ICPL 87 (72%) (Figure 4). Based on the average pod borer complex damage in 39 lines over years, the PSR ranged from 3.0 (PP1505-13-6-3 and PP1505-11-2-5) to 5.0 (PP1501-1-22-3), which showed that these lines were resistant to moderately resistant for pod borer complex (Figure 5). For pod borer (*H. armigera*), 10 ILs were found to be resistant (PSR: 3.0 in PP1501-14-1-5, PP1503-5-2-4, PP1505-63-2-4, PP1505-34-3-6, PP1505-11-2-6, PP1505-13-6-3, PP1505-28-6-1, PP1505-2-8-1, PP1505-11-2-4, and PP1505-11-2-5) to 6 (PP1501-1-22-3) (Figure 5).

### 3.5. Morphological Traits in the Selected ILs Conferring Resistance to Pod Borer

#### 3.5.1. Trichome Density on Adaxial Leaf Surface

The types of trichomes and their range of densities on the adaxial leaf surface of the ILs were as follows: type A, 0–2.7 mm^2^; type B, 15.3–49.7 mm^2^; type C, 8.3–46.7 mm^2^; type D, 1.0–5.7 mm^2^. The mean values on the cultivated recurrent parents were as follows: ICP 8863 (0.7 mm^2^, 16.0 mm^2^, 35.0 mm^2^, and 3.7 mm^2^ for type A, B, C, and D, respectively) and ICPL 87119 (1.3 mm^2^, 57.7 mm^2^, 14.7 mm^2^, and 3.3 mm^2^ for type A, B, C, and D, respectively) (Supplementary Table S8). The highest density of type A trichome was recorded in PP1501-1-18-4 (2.7 mm^2^), that of type B trichomes was in PP1503-5-2-4 (49.7 mm^2^), that of type C trichomes was in PP1501-20-6-2 (46.7 mm^2^), and that of type D trichomes was in PP1501-16-7-2, PP1505-11-2-5, and PP1505-11-2-6 (5.7 mm^2^). The densities of different types of trichomes on the leaves of the checks were as follows: resistant check ICPL 332 WR (2.3 mm^2^, 29.7 mm^2^, 33.0 mm^2^, and 2.3 mm^2^ for type A, B, C, and D trichomes, respectively) and susceptible check ICPL 87 (0.7 mm^2^, 30.0 mm^2^, 14.7 mm^2^, and 1.7 mm^2^ for type A, B, C, and D trichomes, respectively) (Supplementary Table S8).

#### 3.5.2. Trichome Density on Abaxial Leaf Surface

The types of trichomes and their range of densities on the abaxial leaf surface of the ILs were as follows: type A, 0–5.0 mm^2^; type B, 6.0–26.7 mm^2^; type C, 27.3–67.3 mm^2^; type D, 3.3–12.3 mm^2^. The densities of different types of trichomes on the cultivated recurrent parents were as follows: ICP 8863 (0, 12.7 mm^2^, 50.3 mm^2^, and 7.7 mm^2^ for type A, B, C, and D, respectively) and ICPL 87119 (2.3 mm^2^, 27.0 mm^2^, 49.7 mm^2^, and 5.3 mm^2^ for type A, B, C, and D, respectively) (Supplementary Table S8). The highest density of type A trichomes was recorded in PP1501-1-18-4 and PP1503-5-3-3 (5.0 mm^2^), that of type B trichomes was in PP1503-5-3-3 (26.7 mm^2^), that of type C trichomes was in PP1503-25-5-2 (67.3 mm^2^), and that of type D trichomes was in PP1503-20-1-7 (12.3 mm^2^). The densities of different types of trichomes on the leaves of the checks were as follows: resistant check ICPL 332WR (1.3 mm^2^, 17.7 mm^2^, 51.7 mm^2^, and 4.7 mm^2^ for type A, B, C, and D trichomes, respectively) and susceptible check ICPL 87 (0, 14.3 mm^2^, 46.0 mm^2^, and 5.3 mm^2^ for type A, B, C, and D trichomes, respectively) (Supplementary Table S8).

### 3.6. Identification of Promising Pod Borer Resistant ILs

On the basis of the evaluation of the 39 most promising bod borer resistant ILs during the 2020 rainy season and the performance of these 39 ILs in the 2019 rainy season, we identified the most promising ILs showing lower pod damage than that of the respective cultivated recurrent parents and the pod borer resistant checks over the tested years. Based on this criterion, 21 ILs from three populations (11 from PP 1501, two from PP 1503, and eight from PP 1505) with PSR based on pod borer complex and pod borer (*H. armigera*) damage ranging from 3.0 to 4.0 were identified (Table 5; Figure 5). The performance of these ILs across seasons in terms of the levels of pod borer, pod fly, and pod borer complex damage and their biochemical and morphological traits was compared with that of their respective recurrent parents (Table 5). Most of these ILs were also better than the existing pod borer resistant checks. On the basis of these comparisons, the ILs with specific traits related to resistance were identified as candidates for use in pigeonpea improvement programs. These 21 ILs, on an average, took 98–139 days to 50% flowering and 141–180 days to maturity at Warangal. Table 6 lists the best ILs with specific traits related to resistance for ready use in pigeonpea breeding.

## 4. Discussion

Pod borer (*H. armigera*), pod fly (*M. obtusa*), spotted pod borer (*Maruca* *vitrata*), and pod-sucking bug (*C. gibbosa*) can cause grain losses of more than 40% in pigeonpea [2]. Of these, pod borer is the most damaging pest, and it has become strongly resistant to insecticides [19]. Only low to moderate levels of resistance are present in the cultivated germplasm of pigeonpea [20], most of which show >50% pod borer complex damage. Hence, there is a need to exploit the wild relatives of pigeonpea for resistance to this pest. Crop wild relatives (CWR) are important to reintroduce genetic diversity for crop improvement [3]. Wild *Cajanus* species comprise a diverse genetic pool, which can be used to broaden the narrow genetic base of pigeonpea for crop improvement [21,22]. There are different mechanisms conferring resistance to pod borer in wild *Cajanus* accessions, including low density of glandular trichomes (A and B types), high density of non-glandular trichomes (C and D type), antixenosis (oviposition non-preference by insects), antibiosis, and high concentrations of phenols [19]. The wild *Cajanus* species *C. acutifolius* (ICPW 001) and *C. scarabaeoides* (ICPW 281) have been reported to be highly resistant to pod borer, *H. armigera* [9,23]. The pod borer resistance of ICPW 001, which is native to Australia, was reported to be due to high levels of antixenosis and antibiosis [9]. In contrast, the pod borer resistance of ICPW 281, which is native to India, is due to the high density of non-glandular trichomes [19,24]. In the present work, we focused on introgressing pod borer resistance from *C. acutifolius* and *C. scarabaeoides* into two popular pigeonpea cultivars, ICP 8863 (Maruti) and ICPL 87119 (Asha). Here, simple and complex backcross approaches followed by one to two cycles of backcrossing were used to develop four pre-breeding populations. Precise evaluation for pod borer damage over different years and locations was performed. To combine different mechanisms conferring pod borer tolerance from wild *Cajanus* species into the common cultivated background, two complex crosses were generated using ICPW 001 and ICPW 281 as donors in the genetic backgrounds of ICP 8863 and ICPL 87119.

In this study, we evaluated four pre-breeding populations comprising more than 2300 ILs, and then re-evaluated the promising ILs across years and locations. On the basis of these evaluations, we identified 39 ILs with improved resistance to pod borer. These 39 ILs were sourced from three populations: PP 1501 (22 ILs), PP 1503 (6 ILs), and PP 1505 (11 ILs). PP 1503 and PP 1505 were derived from pod borer resistant *C. scarabaeoides* accession ICPW 281 as the pollen donor. The pod borer resistance of ICPW 281 is due to the high density of non-glandular trichomes. All six ILs from PP 1503 and 10 ILs from PP 1505 had high-density type C trichomes, indicating that this trait was successfully introgressed from *C. scarabaeoides* into the pigeonpea cultivar ICPL 87119. Trichome density is negatively associated with larval growth and survival [25,26,27]. In the present study, five ILs from PP 1503 and seven ILs from PP 1505 with a low density of type B trichomes on adaxial and abaxial leaf surfaces and a high density of type C trichomes on the adaxial leaf surface also showed comparatively improved levels of antibiosis (i.e., lower % larval weight gain as compared with those in the susceptible cultivar ICPL 87 and the cultivated recurrent parent ICPL 87119). Based on the level of pod damage (%), two ILs (PP1503-6-1-4 and PP1503-5-2-4) from PP 1503 and eight ILs (PP1505-63-2-4, PP1505-20-5-2, PP1505-36-4-1, PP1505-36-4-2, PP1505-34-3-6, PP1505-11-2-6, PP1505-13-6-3, and PP1505-11-2-5) from PP 1505 showed lower pod borer damage than that of the recipient parent ICPL 87119 both in 2019 and 2020. These ILs also exhibited resistance to pod borer at the Warangal site.

The PP 1501 population was derived from pod borer resistant *C. acutifolius* accession ICPW 001 as the pollen donor. The pod borer resistance of ICPW 001 is associated with a high total flavonoids concentration [28]. Of the 22 ILs from PP 1501, 11 ILs (PP1501-10-17-2, PP1501-12-1-6, PP1501-1-23-3, PP1501-20-6-2, PP1501-4-17-3, PP1501-4-21-6, PP1501-4-17-7, PP1501-14-7-7, PP1501-3-17-3, PP1501-1-10-8, and PP1501-14-1-5) showed lower pod borer damage in both the 2019 and 2020 rainy seasons, as compared to that of the recurrent parent ICP 8863, at Patancheru and Warangal. Except for PP1501-10-17-2 and PP1501-14-1-5, the other nine ILs showed higher total flavonoids concentrations (2.02–6.75 mg/g) than that of the recurrent parent, ICP 8863 (1.67 mg/g). The highest concentrations of phenols and flavonoids were recorded in PP1501-4-17-7 (6.14 mg/g and 6.75 mg/g, respectively), which showed an improved level of antibiosis compared to the cultivated parent, ICP 8863. These results showed that the alleles conferring pod borer resistance were successfully introgressed from *C. acutifolius* into the recurrent cultivated parent ICP 8863. Other studies have also reported that high concentrations of phenols, tannins, and flavonoids confer resistance against pod borer complex [29,30,31,32,33,34]. The pod borer resistance of P1501-10-17-2 may be attributed to the high density of type D trichomes on the adaxial leaf surface, and the high density of type C trichomes on the abaxial leaf surface. In PP1501-14-1-5, pod borer resistance may be due to the high density of type C trichomes on the abaxial leaf surface.

The populations PP 1503 and PP 1504 were developed to combine different mechanisms of pod borer resistance such as high flavonoids and phenols concentrations from *C. acutifolius* and the high density of non-glandular trichomes from *C. scarabaeoides* into the cultivated varieties ICP 8863 and ICPL 87119. Although some ILs in PP 1504 with higher flavonoid concentrations (2.1–3.7 mg/g) than that in the cultivated recurrent parent ICP 8863 (1.67 mg/g) were identified in the 2019 rainy season trial, all these lines had high levels of pod borer complex damage and were not selected for further evaluation. In PP1503, ILs with higher phenols (up to 5.3 mg/g) and flavonoids (12.02 mg/g) concentrations than those in the cultivated recurrent parent ICPL 87119 (2.53 mg/g phenols and 4.58 mg/g flavonoids) were identified, but most of these ILs also had high levels of pod damage due to the large number of insect species that damage pigeonpea (*Helicoverpa armigera, Maruca vitrata, Etiella zincknella, Melanogromyza obtusa*, and others). Only six ILs with low levels of pod damage were selected for further evaluation in 2020. Based on all the traits studied here, only one IL, PP1503-5-2-4, showing low pod damage in both the 2019 and 2020 rainy seasons, had a high total phenols concentration (4.58 mg/g), and a high density of non-glandular type C trichomes on the adaxial leaf surface. This line also showed improved antibiosis for ovipositing insects compared to the cultivated parent ICPL 87119. These results suggest that resistance to pod borers is a complex trait, and that for high concentrations of phenols and/or flavonoids, antibiosis may not be useful as a single criterion to select pod borer resistant ILs. This conclusion is consistent with the findings of other studies [9,35,36,37]. The pod borer resistance of PP1503-6-1-4 may be attributed to the low density of type A and type B trichomes on the adaxial and abaxial leaf surfaces, high density of type C and D trichomes on the adaxial leaf surface and strong antibiosis for larval development.

## 5. Conclusions

The overall results based on pod borer, pod fly, and pod borer complex damage across seasons and biochemical and morphological traits showed that pod borer resistance has been successfully introgressed from *C. acutifolius* and *C. scarabaeoides* into the popular pigeonpea cultivars, ICP 8863 and ICPL 87119. Twenty-one ILs from three populations (11 ILs from PP 1501, two ILs from PP 1503, and eight ILs from PP 1505) showed low pod borer complex damage (< 50% damage) over different years and locations. Because these 21 ILs are more resistant to pod borer than the recipient parents, and their resistance is derived from wild *Cajanus* species, it can be speculated that these ILs contain different alleles related to pod borer resistance. Interestingly, based on the PSR, two ILs (PP1505-13-6-3 and PP1505-11-2-5) were classified as resistant to pod borer complex and 10 ILs (PP1501-14-1-5, PP1503-5-2-4, PP1505-63-2-4, PP1505-34-3-6, PP1505-11-2-6, PP1505-13-6-3, PP1505-28-6-1, PP1505-2-8-1, PP1505-11-2-4, and PP1505-11-2-5) resistant to pod borer. Further, two ILs (PP1501-1-10-8 and PP1503-6-1-4) showing high antibiosis provide novel sources of pod borer resistance for pigeonpea improvement. On the whole, these ILs are potential candidates for the improvement of pod borer resistance in cultivated pigeonpea through gene pyramiding. These ILs represent novel and diverse sources of pod borer resistance for use in large-scale breeding programs for developing new pod borer resistant pigeonpea cultivars.

## Figures and Tables

**Table 1:** Pre-breeding populations developed using wild *Cajanus* species at ICRISAT, Patancheru, India.

Populations	Wild Species Donor	Cross	Generation	No. of ILs
**Simple cross approach**				
PP 1501	*C. acutifolius*	ICP 8863 × (ICP 8863 × (ICP 8863 × ICPW 1))	BC_2_F_3_	1108
PP 1505	*C. scarabaeoides*	ICPL 87119 × (ICPL 87119 × (ICPL 87119 × ICPW 281))	BC_2_F_3_	288
**Complex cross approach**				
PP 1503	*C. acutifolius* and *C. scarabaeoides*	ICPL 87119 × ((ICPL 87119 × ICPW 1) × (ICPL 87119 × ICPW 281))	4-BC_1_F_3_	533
PP 1504	*C. acutifolius* and *C. scarabaeoides*	ICP 8863 × ((ICP 8863 × ICPW 1)× (ICP 8863 × ICPW 281))	4-BC_1_F_3_	392

**Table 2:** Evaluation of four pre-breeding populations derived from wild *Cajanus* species for resistance to pod borer complex during 2018 rainy season at ICRISAT, Patancheru, India. ILs, introgression lines.

S. No.	Populations	No. of ILs	Damage Rating	Recovery Resistance Score	Pod Borer Damage (%)	Pod Fly Damage (%)	Pod Wasp Damage (%)	Plume Moth Damage (%)	Pod Borer Complex Damage (%)	No. of Pod Borer Resistant ILs Identified
1	PP1501	1108	6–9	4–7	7–48	3–55	0–20	7–55	33–85	79
	ICP 8863 (Parent)		8	6	16	38	0	10	60	-
	ENT 11		7	6	29	30	5	7	65	-
	ICPL 332WR		6	6	20	34	4	3	62	-
	ICPL 87119		6	6	20	40	3	7	70	
	ICPL 87		9	7.0	33	44	3	9	75	-
2	PP1503	533	4–9	5–8	6–40	10–44	0–12	0–9	37–80	41
	ICPL 87119 (Parent)		6	7	30	41	2	2	75	-
	ENT 11		6	6	25	30	4	1	60	-
	ICPL 332WR		8	6	26	36	3	1	67	-
	ICPL 87		9	7	30	42	3	2	77	-
3	PP1504	392	6–9	5–9	9–43	22–44	0–8	0–10	46–87	8
	ICP 8863 (Parent)		7	6	25	39	2	1	67	
	ENT 11		7	6	36	44	0	0	67	-
	ICPL 332WR		7	6	25	35	3	2	65	-
	ICPL 87119		7	6	31	39	3	2	74	
	ICPL 87		89	9	45	40	1	1	87	-
4	PP1505	288	6–9	5–8	5–35	7–57	0–8	0–7	23–80	28
	ICPL 87119 (Parent)		7	6	25	37	1	2	63	
	ENT 11		7	6	22	31	2	3	58	-
	ICPL 332WR		7	5	21	30	2	1	54	-
	ICPL 87		9	8	35	33	5	7	80	-

**Table 3:** Re-evaluation of selected introgression lines for resistance to pod borer complex during the 2019 rainy season at ICRISAT, Patancheru, India.

Populations	No. of ILs	Damage Rating	Recovery Resistance Score	Pod Borer Damage (%)	Pod Fly Damage (%)	Pod Wasp Damage (%)	Plume Moth Damage (%)	Pod Borer Complex Damage (%)	No. of Larvae at Podding Stage	No. of Pod Borer Resistant ILs Identified
PP1501	79	6.3 (5–7) *	6.0 (5–7)	20.4 (18–23)	26.9 (11–41)	0.2 (0–1)	2.23 (1–5)	49.73 (32–67)	1.11 (0–2)	22
PP1504	8	6.2 (5–7)	6.1 (5–7)	20.8 (19–25)	29.6 (27–53)	0.26 (0–1)	2.32 (0–4)	52.94 (55–73)	1.12 (1–2)	0
ICP 8863 (Parent)	-	5.0	5. 7	21.0	18.30	0.3	2.30	42.0	0.33	
PP1503	41	6.2 (5–7)	6.1 (5–7)	20.6 (17–25)	27.8 (9–51)	0.25 (0–1)	2.38 (0–7)	51.11 (37–78)	1.14 (0–2)	6
PP1505	28	6.2 (5–7)	6.0 (5–7)	21.3 (16–34)	29.7 (15–46)	0.42 (0–2)	2.41 (1–7)	53.89 (35–72)	1.04 (0–2)	11
ICPL 87119 (Parent)	-	6.3	6.3	21.7	20.0	0.0	4.3	46.0	0.78	
Checks										-
ENT 11		6.33	5.33	18.67	29.00	1.67	1.00	50.33	0.89	
ICPL 332WR		5.33	5.00	18.33	21.67	0.00	4.00	44.00	0.44	
ICPL 87		7.33	7.67	34.33	35.33	0.00	0.67	70.33	2.44	-

* Range given in parentheses.

**Table 4:** Evaluation of 136 selected introgression lines (ILs), parents and checks for resistance to pod borer complex during the 2019 rainy season at Warangal, India.

Populations	No. of ILs	Days to First Flowering	Days to 50% Flowering	Days to Maturity	Recovery Resistance Score	Pod Borer Damage (%)	Spotted Borer Damage (%)	Pod Fly Damage (%)	Pod Wasp Damage (%)	Plume Moth Damage (%)	Pod Borer Complex Damage (%)
PP1501	78	84–131	92–139	135–180	4–8	4–24	2–17	4–32	0–1	0	16–54
PP1504	2	99–108	107–116	145–157	5–7	8–13	6–8	15–24	0	0	34–40
ICP 8863 (Parent)	-	97	105	145	8	9.87	5.4	14.27	0	0	29.53
PP1503	34	96–129	104–137	144–179	4–8	4–25	4–22	3–23	0	0	14–58
PP1505	22	89–131	97–139	140–181	5–7	5–16	3–29	5–24	0	0	22–52
ICPL 87119 (Parent)	-	102	110	161	7	6.3	8.97	19.03	0	0	34.30
Checks											
ENT 11		101	109	155	5.66	8.13	8.93	13.07	0	0	30.13
ICPL 332WR		98	106	146	5.66	4.43	12.07	21.10	0	0	37.60
ICPL 87		73	81	122	7.33	17.60	5.43	5.63	0	0	28.67

**Table 5:** Trait-specific promising introgression lines (ILs) selected from 39 ILs derived from wild *Cajanus* species for use in pigeonpea improvement.

Traits	ILs Better than ICP 8863	ILs Better than ICPL 87119
Low pod borer damage (in both 2019 & 2020)	PP1501-10-17-2, PP1501-12-1-6, PP1501-1-23-3, PP1501-20-6-2, PP1501-4-17-3, PP1501-4-21-6, PP1501-4-17-7, PP1501-14-7-7, PP1501-3-17-3, PP1501-1-10-8 and PP1501-14-1-5	PP1503-6-1-4, PP1503-5-2-4, PP1505-63-2-4, PP1505-20-5-2 PP1505-36-4-1, PP1505-36-4-2, PP1505-34-3-6, PP1505-11-2-6, PP1505-13-6-3 and PP1505-11-2-5
Low pod fly damage (in both 2019 & 2020)	PP1501-1-23-3, PP1501-4-17-3, PP1501-3-17-3	PP1505-13-6-3
Low pod borer complex damage (in both 2019 & 2020)	PP1501-12-1-6, PP1501-1-23-3, PP1501-4-17-3 PP1501-3-17-3	PP1505-34-3-6, PP1505-13-6-3 and PP1505-11-2-5
High phenol content	PP1501-4-17-7 (6.14 mg/g)	PP1503-5-2-4 (4.58 mg/g) PP1505-63-2-4 (3.98 mg/g) PP1505-11-2-5 (6.99 mg/g)
High flavonoid content	PP1501-12-1-6, PP1501-1-23-3, PP1501-20-6-2, PP1501-4-17-3, PP1501-4-21-6, PP1501-4-17-7, PP1501-14-7-7, PP1501-3-17-3, PP1501-1-10-8 (2.02–6.75 mg/g)	PP1505-11-2-5 (7.94 mg/g)
Antibiosis	PP1501-10-17-2, PP1501-12-1-6, PP1501-20-6-2, PP1501-4-17-7, PP1501-14-7-7, PP1501-3-17-3, PP1501-1-10-8 and PP1501-14-1-5 (45.1–609.9% larval weight gain)	PP1503-6-1-4, PP1505-11-2-5, PP1503-5-2-4, PP1505-20-5-2, PP1505-36-4-1, PP1505-36-4-2, PP1505-34-3-6, PP1505-11-2-6 and PP1505-13-6-3 (163.5–367.9% larval weight gain)
Low density of type A trichomes on adaxial leaf surface	PP1501-1-23-3, PP1501-20-6-2, PP1501-1-10-8 PP1501-14-1-5	PP1503-6-1-4, PP1505-63-2-4, PP1505-36-4-1, PP1505-36-4-2, PP1505-34-3-6, PP1505-11-2-6 and PP1505-13-6-3
Low density of type B trichomes on adaxial leaf surface	-	PP1503-6-1-4, PP1503-5-2-4, PP1505-63-2-4, PP1505-20-5-2, PP1505-36-4-1, PP1505-36-4-2, PP1505-34-3-6, PP1505-11-2-6, PP1505-11-2-5 and PP1505-13-6-3
High density of type C trichomes on adaxial leaf surface	PP1501-20-6-2, PP1501-1-10-8	PP1503-6-1-4, PP1503-5-2-4, PP1505-63-2-4, PP1505-20-5-2, PP1505-36-4-1, PP1505-36-4-2, PP1505-34-3-6, PP1505-11-2-6, PP1505-11-2-5 and PP1505-13-6-3
High density of type D trichomes on adaxial leaf surface	PP1501-20-6-2, PP1501-1-10-8, PP1501-10-17-2 PP1501-12-1-6	PP1505-63-2-4, PP1505-11-2-6 and PP1505-11-2-5
Low density of type A trichomes on abaxial leaf surface	PP1501-4-17-3, PP1501-1-10-8	PP1503-6-1-4, PP1503-5-2-4, PP1505-63-2-4, PP1505-20-5-2, PP1505-36-4-1, PP1505-36-4-2, PP1505-34-3-6, PP1505-11-2-6, PP1505-11-2-5 and PP1505-13-6-3
Low density of type B trichomes on abaxial leaf surface	PP1501-1-23-3	PP1503-6-1-4, PP1503-5-2-4, PP1505-63-2-4, PP1505-20-5-2, PP1505-36-4-1, PP1505-36-4-2, PP1505-34-3-6, PP1505-11-2-6, PP1505-11-2-5 and PP1505-13-6-3
High density of type C trichomes on abaxial leaf surface	PP1501-10-17-2, PP1501-12-1-6, PP1501-20-6-2 PP1501-14-1-5	PP1505-63-2-4, PP1505-36-4-1, PP1505-11-2-6 and PP1505-11-2-5
High density of type D trichomes on abaxial leaf surface	PP1501-14-7-7	PP1505-63-2-4, PP1505-20-5-2, PP1505-36-4-1, PP1505-36-4-2, PP1505-34-3-6 and PP1505-11-2-5

**Table 6:** Details of the most promising pod borer-tolerant introgression lines that could be utilized in pigeonpea improvement programs.

Introgression Line	Key Traits
PP1505-63-2-4	Low pod borer (~14%) and pod borer complex damage (~44%), high phenols (3.98 mg/g) and flavonoids (3.46 mg/g) content, low density of trichomes A and B, and high density of trichomes C and D on leaves
PP1505-13-6-3	Low pod borer (~17%), pod fly (~14%), and pod borer complex (~35%) damage, low density of trichomes A and B and high density of trichome C on leaves, phenols (1.97 mg/g) and flavonoids (2.76 mg/g), and resistant to pod borer and pod borer complex (based on PSR 3.0)
PP1505-11-2-5	Low pod borer (~18%), pod fly (~16%), and pod borer complex (~38%) damage, high phenols (6.99 mg/g) and flavonoids (7.94 mg/g) conten, low density of trichomes A and B and high density of trichomes C and D on leaves, and resistant to pod borer and pod borer complex (based on PSR 3.0)
PP1503-6-1-4	Low pod borer (19%) and pod borer complex damage (~45%), high antibiosis (163.5% larval weight gain), low density of trichomes A and B and high density of trichome C on leaves, phenols (1.34 mg/g), and flavonoids (2.33 mg/g)
PP1503-5-2-4	Low pod borer damage (18.5%), low density of trichomes A and B and high density of trichome C on leaves, high phenols (4.58 mg/g), and flavonoids (2.29 mg/g)
PP1501-1-10-8	Low pod borer (~18%) and pod borer complex damage (~47%), high antibiosis (45% larval weight gain), low density of trichome A and high density of trichomes C and D on leaves, phenols (2.01 mg/g), and high flavonoids (2.63 mg/g)
PP1501-4-17-7	Low pod borer damage (18%), high phenols (6.14 mg/g), and high flavonoids (6.75 mg/g)

## Data Availability

All data relevant to this study are provided in the manuscript and Supplementary Materials.

## Supplementary Materials

The following supporting information can be downloaded at: https://www.mdpi.com/article/10.3390/biology11040485/s1. Supplementary Table S1: List of introgression lines (ILs) selected from the evaluation of advanced backcross population, PP 1501, derived from *Cajanus acutifolius* for pod borer complex damage during the 2018 rainy season at ICRISAT, Patancheru, India. Supplementary Table S2: List of introgression lines selected from the evaluation of advanced backcross population, PP 1503, derived from the complex cross for pod borer complex damage during the 2018 rainy season at ICRISAT, Patancheru, India. Supplementary Table S3: List of introgression lines selected from the evaluation of advanced backcross population, PP 1504, derived from the complex cross for pod borer complex damage during the 2018 rainy season at ICRISAT, Patancheru, India. Supplementary Table S4: List of introgression lines selected from the evaluation of advanced backcross population, PP 1505, derived from *Cajanus scarabaeoides* for pod borer complex damage during the 2018 rainy season at ICRISAT, Patancheru, India. Supplementary Table S5: Evaluation of selected introgression lines derived from wild *Cajanus* species for resistance to pod borer complex during the 2019 rainy season at ICRISAT, Patancheru, India. Supplementary Table S6: Detached pod assay of the selected introgression lines for pod borer resistance under laboratory conditions during the 2019 rainy season at ICRISAT, Patancheru, India. Supplementary Table S7: Evaluation of introgression lines for pod borer damage and important agronomic traits during the 2019 rainy season at Warangal, India. Supplementary Table S8: Trichome density in the 39 most promising pod borer resistant introgression lines, as well as parents and checks, during the 2020 rainy season at ICRISAT, Patancheru, India.
