# Supplementary material for: Reaping the Potential of Wild Cajanus Species through Pre-Breeding for Improving Resistance to Pod Borer, Helicoverpa armigera, in Cultivated Pigeonpea (Cajanus cajan (L.) Millsp.)
Source: Biology (Basel). 2022 Mar 22;11(4):485. doi: 10.3390/biology11040485 (PMC9026160; doi:10.3390/biology11040485)
Supplement: Supplementary file 1 [file biology-11-00485-s001.zip › biology-1598577-supplementary.pdf]

Supplementary Table S1. List of introgression lines (ILs) selected from the evaluation of advanced backcross population, PP 1501, derived from *Cajanus acutifolius* for pod borer complex damage during the 2018 rainy season at ICRISAT, Patancheru, India.

S. No.	Genotype identity	Damage rating	Recovery resistance score	Total no. of pods	Healthy pods (%)	Pod borer damage (%)	Pod fly damage (%)	Pod wasp damage (%)	Plume moth damage (%)	Total pod borer complex damage (%)
1	PP1501-1-2-2	-	6	100	34	10	37	17	2	66
2	PP1501-1-2-4	6	5	100	48	25	23	4	0	52
3	PP1501-1-5-2	7	6	100	45	30	14	0	11	55
4	PP1501-1-6-2	8	6	100	56	21	20	0	3	44
5	PP1501-1-8-2	7	6	100	37	12	35	1	15	63
6	PP1501-1-10-1	7	5	100	58	20	16	0	6	42
7	PP1501-1-10-4	7	5	100	49	20	30	1	0	51
8	PP1501-1-10-8	7	5	100	54	15	30	1	0	46
9	PP1501-1-12-3	8	5	100	47	9	38	4	2	53
10	PP1501-1-12-8	7	5	100	25	30	35	0	10	75
11	PP1501-1-13-2	7	5	100	32	28	26	6	8	68
12	PP1501-1-13-3	7	5	100	28	32	21	11	8	72
13	PP1501-1-13-4	7	5	100	50	26	23	1	0	50
14	PP1501-1-13-5	7	4	100	65	23	10	2	0	35
15	PP1501-1-18-4	8	6	100	55	15	30	0	0	45
16	PP1501-1-20-2	8	5	100	30	32	31	4	3	70
17	PP1501-1-20-6	7	5	100	44	30	17	0	9	56
18	PP1501-1-22-1	8	6	100	36	14	25	20	5	64
19	PP1501-1-22-2	8	5	100	38	31	26	1	4	62
20	PP1501-1-22-3	9	5	100	34	34	26	5	1	66
21	PP1501-1-22-4	8	5	100	20	33	34	5	8	80
22	PP1501-1-22-5	7	6	100	32	34	12	8	14	68

23	PP1501-1-23-2	9	5	100	50	20	10	8	12	50
24	PP1501-1-23-3	8	5	100	27	23	38	6	6	73
25	PP1501-2-2-5	8	6	100	54	14	26	0	6	46
26	PP1501-2-3-6	8	6	100	59	12	26	1	2	41
27	PP1501-2-4-1	7	5	100	22	30	35	5	8	78
28	PP1501-2-5-1	8	6	100	38	10	48	2	2	62
29	PP1501-2-6-2	7	6	100	59	25	13	2	1	41
30	PP1501-2-9-4	8	6	100	50	15	32	3	0	50
31	PP1501-2-13-2	8	6	100	42	14	34	10	0	58
32	PP1501-2-16-4	7	6	100	40	41	14	2	3	60
33	PP1501-3-11-2	7	7	100	27	23	43	1	6	73
34	PP1501-3-15-1	8	6	100	28	15	53	4	0	72
35	PP1501-3-17-3	8	6	100	55	18	20	2	5	45
36	PP1501-4-8-2	7	5	100	18	40	32	8	2	82
37	PP1501-4-9-2	8	5	100	35	30	35	0	0	65
38	PP1501-4-9-3	8	5	100	25	23	39	9	4	75
39	PP1501-4-12-1	8	5	100	19	48	25	4	4	81
40	PP1501-4-12-2	8	5	100	30	25	40	0	5	70
41	PP1501-4-12-4	8	5	100	40	26	29	2	3	60
42	PP1501-4-12-5	-	5	100	16	25	51	4	4	84
43	PP1501-4-14-3	8	5	100	35	28	32	2	3	65
44	PP1501-4-14-4	8	5	100	40	24	30	3	3	60
45	PP1501-4-17-1	8	5	100	38	25	30	4	3	62
46	PP1501-4-17-2	8	5	100	25	34	36	0	5	75
47	PP1501-4-17-3	8	5	100	38	30	32	0	0	62
48	PP1501-4-17-7	8	5	100	40	20	30	8	2	60
49	PP1501-4-20-1	8	5	100	45	16	34	2	3	55
50	PP1501-4-21-6	8	6	100	56	29	14	1	0	44
51	PP1501-5-14-1	8	5	100	20	35	38	4	3	80

52	PP1501-5-14-3	-	5	100	28	21	45	3	5	74
53	PP1501-5-14-4	-	5	100	15	20	55	5	5	85
54	PP1501-5-15-3	-	5	100	23	32	38	2	5	77
55	PP1501-5-15-7	-	6	100	37	15	44	0	4	63
56	PP1501-6-12-2	8	5	100	18	27	49	4	2	82
57	PP1501-7-19-5	-	5	100	54	20	23	0	3	46
58	PP1501-9-9-2	8	5	100	35	25	35	3	2	65
59	PP1501-9-18-1	8	6	100	20	30	3	2	55	35
60	PP1501-10-17-1	8	6	100	50	31	13	1	5	50
61	PP1501-10-17-2	8	6	100	49	34	13	2	2	51
62	PP1501-12-1-1	7	4	100	25	38	25	4	8	75
63	PP1501-12-1-2	8	4	100	57	21	14	2	6	43
64	PP1501-12-1-3	8	5	100	35	25	30	8	2	65
65	PP1501-12-1-4	7	5	100	52	16	24	2	6	48
66	PP1501-12-1-5	-	5	100	30	25	30	5	10	70
67	PP1501-12-1-6	8	5	100	40	33	23	2	2	60
68	PP1501-12-1-7	8	5	100	40	24	34	0	2	60
69	PP1501-14-1-5	8	5	100	57	14	24	1	4	43
70	PP1501-14-2-1	7	6	100	45	15	38	0	2	55
71	PP1501-14-2-3	8	5	100	45	20	30	1	4	55
72	PP1501-14-4-3	-	6	100	48	7	35	5	5	52
73	PP1501-14-5-3	7	5	100	50	20	25	2	3	50
74	PP1501-14-7-7	8	6	100	61	22	15	0	2	39
75	PP1501-16-6-5	-	5	100	35	25	35	2	3	65
76	PP1501-16-7-2	8	6	100	62	7	24	3	4	38
77	PP1501-16-7-6	8	6	100	67	7	22	1	3	33
78	PP1501-16-7-7	7	5	100	40	20	28	10	2	60
79	PP1501-20-6-2	-	5	100	42	33	22	1	2	58
80	ICP 8863	8	6	100	40	16	38	0	10	65

	(Recurrent parent)									
81	ENT 11	7	6	100	29	29	30	5	7	71
82	ICPL 332WR	6	5	100	38	20	34	4	3	62
83	ICPL 87119	6	6	100	30	20	40	3	7	70
84	ICPL 88039	8	8	100	39	33	22	5	1	61
85	ICP 7035	7	6	100	41	25	27	3	4	59
86	ICPL 85010	9	9	100	40	29	29	0	2	60
87	ICPL 87	9	7	100	25	33	44	3	9	75

Supplementary Table S2: List of introgression lines selected from the evaluation of advanced backcross population, PP 1503, derived from the complex cross for pod borer complex damage during the 2018 rainy season at ICRISAT, Patancheru, India.

S. No.	Genotype identity	Damage rating	Recovery resistance score	Total no. of pods	Healthy pods (%)	Pod borer damage (%)	Pod fly damage (%)	Pod wasp damage (%)	Plume moth damage (%)	Total pod borer complex damage (%)
1	PP1503-3-1-3	-	6	**100**	51	11	35	2	1	49
2	PP1503-5-2-4	8	5	**100**	61	14	14	2	9	39
3	PP1503-5-3-1	8	5	**100**	47	20	31	0	2	53
4	PP1503-5-3-3	8	6	**100**	55	20	24	1	0	45
5	PP1503-6-1-4	8	6	**100**	61	21	16	0	2	39
6	PP1503-6-2-5	7	5	**100**	45	25	25	3	2	55
7	PP1503-8-2-4	8	5	**100**	45	25	30	0	0	55
8	PP1503-8-3-2	-	5	**100**	25	25	40	8	2	75
9	PP1503-10-5-4	7	6	**100**	35	27	38	0	0	65
10	PP1503-10-5-6	-	6	**100**	35	20	35	6	4	65
11	PP1503-11-1-1	9	6	**100**	53	6	38	1	2	47
12	PP1503-11-1-4	7	6	**100**	59	21	20	0	0	41
13	PP1503-12-1-7	8	5	**100**	40	28	32	0	0	60
14	PP1503-12-2-2	7	5	**100**	20	35	36	5	4	80
15	PP1503-12-2-6	7	5	**100**	35	33	25	4	3	65
16	PP1503-14-2-7	8	5	**100**	32	30	35	2	1	68
17	PP1503-15-1-2	-	5	**100**	39	20	33	4	4	61
18	PP1503-15-1-7	-	5	**100**	30	30	35	3	2	70
19	PP1503-15-2-2	-	5	**100**	30	30	35	3	2	70
20	PP1503-15-3-3	8	5	**100**	38	30	32	0	0	62
21	PP1503-15-3-4	-	5	**100**	41	22	31	3	3	59
22	PP1503-16-3-8	8	5	**100**	30	40	20	5	5	70

23	PP1503-18-1-7	7	6	**100**	57	16	25	2	0	43
24	PP1503-20-1-2	8	5	**100**	38	20	30	8	4	62
25	PP1503-20-1-5	8	5	**100**	35	25	35	3	2	65
26	PP1503-22-1-5	8	6	**100**	45	40	12	1	2	55
27	PP1503-20-1-7	8	5	**100**	39	20	31	5	5	61
28	PP1503-22-3-1	-	6	**100**	56	18	23	3	0	44
29	PP1503-22-3-3	7	5	**100**	30	30	35	3	2	70
30	PP1503-22-4-5	8	5	**100**	30	30	35	5	0	70
31	PP1503-25-4-4	7	5	**100**	45	20	30	3	2	55
32	PP1503-25-5-2	8	5	**100**	45	25	28	2	0	55
33	PP1503-25-5-6	7	6	**100**	63	19	18	0	0	37
34	PP1503-26-3-5	6	5	**100**	30	35	28	4	3	70
35	PP1503-27-2-8	-	5	**100**	46	19	21	5	9	54
36	PP1503-27-4-4	6	5	**100**	20	30	40	5	5	80
37	PP1503-28-1-3	7	5	**100**	35	20	38	4	3	65
38	PP1503-29-1-5	-	6	**100**	60	30	10	0	0	40
39	PP1503-29-2-1	7	6	**100**	50	15	35	0	0	50
40	PP1503-29-2-7	-	5	**100**	44	28	20	0	8	56
41	PP1503-30-1-6	6	5	**100**	30	28	32	8	2	70
42	ICPL 87119 (Recurrent parent)	6	7	**100**	0	0	0	0	0	0
43	ENT 11	6	6	**100**	39	25	30	4	1	60
44	ICPL 332WR	6	5	**100**	33	26	36	3	1	67
45	ICPL 87119	6	6	**100**	25	30	41	2	2	75
46	ICPL 88039	7	7	**100**	27	27	41	4	1	72
47	ICP 7035	4	5	**100**	29	31	35	2	1	71
48	ICPL 85010	8	8	**100**	20	29	44	4	3	80
49	ICPL 87	9	8	**100**	24	30	42	3	2	77

Supplementary Table S3. List of introgression lines selected from the evaluation of advanced backcross population, PP 1504, derived from the complex cross for pod borer complex damage during the 2018 rainy season at ICRISAT, Patancheru, India.

S. No.	Genotype identity	Damage rating	Recovery resistance score	Total no. of pods	Healthy pods (%)	Pod borer damage (%)	Pod fly damage (%)	Pod wasp damage (%)	Plume moth damage (%)	Total pod borer complex damage (%)
**1**	PP1504-1-3-1	7	5	100	40	20	29	4	7	**60**
**2**	PP1504-2-1-3	8	6	100	46	17	33	2	2	**54**
**3**	PP1504-2-2-1	8	5	100	30	30	30	5	5	**70**
**4**	PP1504-2-11-5	8	6	100	50	22	26	1	1	**50**
**5**	PP1504-3-7-2	-	6	100	45	20	26	5	0	**51**
**6**	PP1504-4-8-2	8	6	100	53	9	37	0	1	**47**
**7**	PP1504-4-9-1	-	6	100	54	11	35	0	0	**46**
**8**	PP1504-4-9-2	-	6	100	32	13	35	3	1	**52**
**9**	ICP 8863 (Recurrent parent)	7	6	100	23	25	39	2	1	**67**
**10**	ENT 11	7	6	100	20	36	44	0	0	**67**
**11**	ICPL 332WR	7	6	100	35	25	35	3	1	**65**
**12**	ICPL 87119	7	6	100	26	31	39	3	1	**74**
**13**	ICPL 88039	7	7	100	20	25	50	3	2	**80**
**14**	ICP 7035	6	6	100	27	29	38	4	2	**73**
**15**	ICPL 85010	9	9	100	-	-	-	-	-	**-**
**16**	ICPL 87	8	9	100	13	45	40	1	1	**87**

Supplementary Table S4. List of introgression lines selected from the evaluation of advanced backcross population, PP 1505, derived from *Cajanus scarabaeoides* for pod borer complex damage during the 2018 rainy season at ICRISAT, Patancheru, India.

S. No	Genotype identity	Damage rating	Recovery resistance score	Total no. of pods	Healthy pods (%)	Pod borer damage (%)	Pod fly damage (%)	Pod wasp damage (%)	Plume moth damage (%)	Total pod borer complex damage (%)
1	PP1505-4-4-1	8	5	100	35	30	30	3	2	65
2	PP1505-32-3-2	7	5	100	48	24	24	2	2	52
3	PP1505-32-5-3	-	5	100	35	30	30	3	2	65
4	PP1505-59-4-1	-	5	100	40	20	30	5	5	60
5	PP1505-59-4-3	7	5	100	40	16	35	3	6	60
6	PP1505-59-4-4	7	5	100	58	10	23	3	6	42
7	PP1505-59-4-6	7	5	100	35	25	30	4	6	65
8	PP1505-59-7-1	7	5	100	39	25	26	5	5	61
9	PP1505-59-7-2	7	5	100	49	21	18	5	7	51
10	PP1505-2-3-1	6	6	100	60	25	15	0	0	40
11	PP1505-2-8-1	8	6	100	50	12	36	2	0	50
12	PP1505-11-2-2	8	6	100	57	28	14	0	1	43
13	PP1505-11-2-4	7	6	100	64	8	26	1	1	36
14	PP1505-11-2-5	-	6	100	72	10	18	0	0	28
15	PP1505-11-2-6	-	6	100	77	11	7	5	0	23
16	PP1505-13-6-3	8	6	100	64	18	11	7	0	36
17	PP1505-20-5-2	-	6	100	56	16	22	5	1	44
18	PP1505-28-6-1	8	6	100	73	11	15	0	1	27
19	PP1505-29-4-1	7	6	100	55	20	24	1	0	45
20	PP1505-31-1-2	8	6	100	55	15	28	2	0	45
21	PP1505-34-3-6	7	6	100	62	5	32	1	0	38
22	PP1505-36-4-1	-	6	100	56	15	28	1	0	44

23	PP1505-36-4-2	-	6	100	58	18	23	0	1	42
24	PP1505-36-4-4	7	6	100	54	12	28	2	4	46
25	PP1505-61-4-1	7	6	100	47	9	42	1	1	53
26	PP1505-63-2-4	-	6	100	61	7	30	2	0	39
27	PP1505-63-2-5	-	6	100	52	22	26	0	0	48
28	PP1505-63-2-6	-	6	100	22	12	57	6	3	78
29	ICPL 87119 (Recurrent parent)	7	6	100	37	25	37	1	2	63
30	ENT 11	7	6	100	43	22	31	2	3	58
31	ICPL 332	7	5	100	46	21	30	2	1	54
32	ICPL 88039	8	8	100	40	28	23	3	6	60
33	ICP 7035	6	6	100	38	35	26	0	1	62
34	ICPL 85010	9	8	-	-	-	-	-	-	-
35	ICPL 87	9	8	100	20	35	33	5	7	80

Supplementary Table S5. Evaluation of selected introgression lines derived from wild *Cajanus* species for resistance to pod borer complex during the 2019 rainy season at ICRISAT, Patancheru, India.

S. No.	Genotype identity	Damage rating	Recovery resistance score	Total no. of pods	Healthy pods (%)	Pod borer damage (%)	Pod fly damage (%)	Pod wasp damage (%)	Plume moth damage (%)	Total pod borer complex damage (%)	Number of larvae (*H. armigera*) at podding stage
1	PP 1501 -14-5-3	6.67	6.00	100	38.33	21.67	39.00	0.00	1.00	61.67	1.22
2	PP1501-1-10-1	6.00	5.67	100	47.33	21.67	29.00	0.00	2.00	52.67	1.0
3	PP1501-1-10-4	6.33	5.33	100	49.00	23.00	23.00	0.67	4.33	51.00	1.22
4	PP1501-1-10-8	5.67	5.33	100	50.00	19.33	28.67	0.33	1.67	50.00	0.11
5	PP1501-1-12-3	5.67	6.00	100	36.67	22.67	37.00	0.00	3.67	63.33	1.67
6	PP1501-1-12-8	5.00	5.00	100	36.33	20.67	38.67	0.00	4.33	63.67	1.22
7	PP1501-1-13-2	6.67	6.33	100	60.00	22.67	14.67	0.33	2.33	40.00	0.89
8	PP1501-1-13-3	7.00	6.33	100	44.33	17.67	37.33	0.33	0.33	55.67	1.33
9	PP1501-1-13-4	7.33	5.67	100	58.33	18.67	21.33	0.00	1.67	41.67	1.0
10	PP1501-1-13-5	5.67	5.33	100	59.67	22.00	15.33	0.33	2.67	40.33	0.78
11	PP1501-1-18-4	6.33	6.33	100	54.33	21.33	22.00	0.00	2.33	45.67	1.22
12	PP1501-1-2-2	6.67	6.67	100	37.00	19.67	40.00	1.33	2.00	63.00	1.44
13	PP1501-1-2-4	5.00	6.00	100	38.67	18.67	41.33	0.33	1.00	61.33	1.33
14	PP1501-1-20-2	6.33	6.67	100	36.00	20.67	39.33	0.33	3.67	64.00	1.33
15	PP1501-1-20-6	6.67	6.33	100	58.33	21.00	19.00	0.00	1.67	41.67	1.22
16	PP1501-1-22-1	7.00	7.33	100	52.67	23.33	22.00	0.33	1.67	47.33	1.08
17	PP1501-1-22-2	6.67	6.00	100	54.67	21.00	22.67	0.33	1.33	45.33	1.33
18	PP1501-1-22-3	6.67	7.00	100	67.00	21.33	9.33	0.00	2.33	33.00	0.89
19	PP1501-1-22-4	6.33	6.33	100	57.33	21.33	15.67	0.67	5.00	42.67	1.67
20	PP1501-1-22-5	5.67	5.67	100	55.33	19.67	23.00	1.33	0.67	44.67	1.67

21	PP1501-1-23-2	5.67	5.00	100	60.00	22.67	14.67	0.00	2.67	40.00	0.78
22	PP1501-1-23-3	5.67	5.67	100	60.33	20.67	16.67	0.00	2.33	39.67	0.56
23	PP1501-1-5-2	6.67	6.33	100	47.00	20.33	30.00	0.67	2.00	53.00	1.33
24	PP1501-1-6-2	6.00	6.00	100	55.33	22.00	21.33	0.00	1.33	44.67	0.89
25	PP1501-1-8-2	6.00	6.00	100	55.00	18.00	26.50	0.00	0.50	45.00	1.78
26	PP1501-10-17-1	7.00	7.00	100	49.33	20.67	26.33	0.00	3.67	50.67	1.11
27	PP1501-10-17-2	5.67	6.00	100	58.00	18.67	22.00	0.00	1.33	42.00	0.67
28	PP1501-12-1-1	5.67	5.67	100	42.67	20.33	35.33	0.00	1.67	57.33	1.56
29	PP1501-12-1-2	5.33	5.67	100	48.67	20.00	30.33	0.00	1.00	51.33	0.11
30	PP1501-12-1-3	6.33	5.67	100	54.00	19.33	26.00	0.00	0.67	46.00	1.33
31	PP1501-12-1-4	6.00	6.33	100	47.00	19.00	31.67	0.33	2.00	53.00	1.66
32	PP1501-12-1-5	6.00	5.67	100	59.33	18.33	21.00	0.33	1.00	40.67	1.0
33	PP1501-12-1-6	5.33	5.00	100	58.33	20.67	20.33	0.00	0.67	41.67	0.44
34	PP1501-12-1-7	6.33	5.67	100	48.67	20.33	29.33	0.00	1.67	51.33	1.22
35	PP1501-14-1-5	6.00	5.33	100	52.00	18.67	23.33	0.00	6.00	48.00	0.67
36	PP1501-14-2-1	6.00	5.67	100	31.00	23.67	41.00	0.00	4.33	69.00	1.22
37	PP1501-14-2-3	6.67	6.33	100	42.00	21.00	34.67	0.33	2.00	58.00	1.11
38	PP1501-14-4-3	5.33	6.00	100	40.33	22.67	33.67	0.67	2.67	59.67	1.56
39	PP1501-14-7-7	5.67	5.33	100	56.33	19.67	21.67	0.00	2.33	43.67	0.67
40	PP1501-16-6-5	6.00	6.00	100	43.67	22.33	32.00	0.00	2.00	56.33	1.22
41	PP1501-16-7-2	6.00	5.33	100	53.33	22.00	21.00	0.00	3.67	46.67	0.22
42	PP1501-16-7-6	6.33	6.00	100	42.33	19.00	36.00	1.00	1.67	57.67	1.08
43	PP1501-16-7-7	6.33	5.00	100	51.33	24.00	18.67	0.33	5.67	48.67	1.67
44	PP1501-2-13-2	7.00	6.67	100	45.00	19.67	33.33	1.00	1.00	55.00	1.67
45	PP1501-2-16-4	6.33	6.33	100	59.00	19.33	19.00	0.00	2.67	41.00	0.99
46	PP1501-2-2-5	6.33	7.00	100	37.67	21.33	39.67	0.00	1.33	62.33	1.11
47	PP1501-2-3-6	5.67	6.33	100	67.67	18.00	12.33	0.33	1.67	32.33	0.33
48	PP1501-2-4-1	6.33	6.33	100	51.33	20.00	27.00	0.33	1.33	48.67	1.33
49	PP1501-2-5-1	6.67	6.33	100	48.33	18.33	31.00	0.00	2.33	51.67	1.66

50	PP1501-2-6-2	5.67	6.00	100	32.33	24.00	36.33	0.33	7.00	67.67	1.22
51	PP1501-2-9-4	6.33	6.67	100	59.33	19.67	15.33	0.00	5.67	40.67	0.89
52	PP1501-20-6-2	6.00	5.67	100	49.67	20.00	26.33	0.00	4.00	50.33	0.62
53	PP1501-3-11-2	6.00	6.00	100	63.00	20.33	14.67	0.00	2.00	37.00	1.33
54	PP1501-3-15-1	7.00	6.33	100	39.67	18.67	38.67	0.33	2.67	60.33	1.19
55	PP1501-3-17-3	6.33	6.33	100	66.67	20.00	11.33	0.33	1.67	33.33	0.67
56	PP1501-4-12-1	6.33	5.67	100	56.33	18.00	25.33	0.00	0.33	43.67	1.22
57	PP1501-4-12-2	6.33	6.33	100	60.67	17.67	19.00	0.67	2.00	39.33	2.0
58	PP1501-4-12-4	6.33	5.67	100	53.33	22.33	21.33	0.33	2.67	46.67	1.33
59	PP1501-4-12-5	6.33	5.67	100	61.67	18.00	19.33	0.00	1.00	38.33	1.08
60	PP1501-4-14-3	6.33	5.67	100	42.67	22.67	31.67	0.33	2.67	57.33	1.33
61	PP1501-4-14-4	6.33	6.67	100	41.33	20.33	36.00	0.00	2.33	58.67	1.33
62	PP1501-4-17-1	5.67	6.00	100	58.67	21.67	15.67	0.00	4.00	41.33	2.0
63	PP1501-4-17-2	7.00	6.67	100	58.67	19.33	21.00	0.33	0.67	41.33	1.0
64	PP1501-4-17-3	6.33	6.00	100	62.33	19.33	17.33	0.00	1.00	37.67	0.33
65	PP1501-4-17-7	6.67	6.67	100	53.33	18.00	27.67	0.33	0.67	46.67	0.11
66	PP1501-4-20-1	7.00	6.67	100	56.33	16.67	23.67	2.00	1.33	43.67	1.33
67	PP1501-4-21-6	6.67	6.33	100	52.67	20.00	24.67	0.00	2.67	47.33	0.89
68	PP1501-4-8-2	7.00	6.33	100	42.00	18.67	38.00	1.00	0.33	58.00	1.56
69	PP1501-4-9-2	7.00	6.67	100	49.33	19.00	28.67	0.33	2.67	50.67	1.44
70	PP1501-4-9-3	7.00	6.67	100	46.50	21.00	29.50	0.00	3.00	53.50	1.22
71	PP1501-5-14-1	6.67	5.67	100	36.67	19.33	41.33	0.33	2.33	63.33	1.33
72	PP1501-5-14-3	6.33	5.67	100	53.33	21.00	23.33	0.00	2.33	46.67	0.11
73	PP1501-5-14-4	5.33	5.33	100	46.33	20.33	32.33	0.00	1.00	53.67	0.99
74	PP1501-5-15-3	6.67	6.33	100	48.67	19.67	28.33	0.00	3.33	51.33	1.11
75	PP1501-5-15-7	6.00	6.00	100	48.33	21.33	27.00	0.00	3.33	51.67	1.08
76	PP1501-6-12-2	6.33	5.00	100	45.50	20.00	32.50	0.50	1.50	54.50	0.99
77	PP1501-7-19-5	6.67	6.33	100	46.00	23.67	28.67	0.67	1.00	54.00	1.22
78	PP1501-9-18-1	6.67	6.67	100	41.67	19.67	37.33	0.67	0.67	58.33	1.22

79	PP1501-9-9-2	6.33	5.67	100	42.00	21.00	34.67	0.33	2.00	58.00	1.11
80	PP1503-10-5-4	5.67	5.67	100	40.67	24.67	31.00	1.33	2.33	59.33	1.0
81	PP1503-10-5-6	6.67	6.00	100	47.67	20.33	28.00	0.00	4.00	52.33	1.08
82	PP1503-11-1-1	6.33	6.00	100	30.33	21.67	47.00	0.00	1.00	69.67	1.08
83	PP1503-11-1-4	5.67	7.00	100	41.00	17.33	40.33	0.33	1.00	59.00	0.99
84	PP1503-12-1-7	5.33	6.00	100	42.67	20.33	31.33	0.00	5.67	57.33	1.33
85	PP1503-12-2-2	6.00	6.00	100	40.67	22.33	33.00	1.00	3.00	59.33	1.08
86	PP1503-12-2-6	7.00	6.33	100	51.67	20.67	22.33	0.67	4.67	48.33	1.22
87	PP1503-14-2-7	6.33	6.00	100	41.67	20.67	33.00	0.00	4.67	58.33	1.33
88	PP1503-15-1-2	6.33	5.33	100	52.67	20.00	25.67	0.00	1.67	47.33	1.2
89	PP1503-15-1-7	5.67	6.33	100	39.67	22.67	35.67	0.33	1.67	60.33	1.22
90	PP1503-15-2-2	6.00	6.33	100	59.67	19.00	21.00	0.33	0.00	40.33	1.33
91	PP1503-15-3-3	5.67	5.67	100	50.33	21.33	26.67	0.33	1.33	49.67	1.44
92	PP1503-15-3-4	6.00	5.67	100	49.00	18.67	31.67	0.00	0.67	51.00	1.22
93	PP1503-16-3-8	6.00	6.33	100	54.33	19.67	23.00	0.33	2.67	45.67	0.11
94	PP1503-18-1-7	6.00	6.33	100	32.00	22.67	43.67	0.33	1.33	68.00	1.11
95	PP1503-20-1-2	6.00	5.67	100	50.00	18.00	29.33	0.00	2.67	50.00	1.80
96	PP1503-20-1-5	5.33	6.00	100	51.67	25.00	19.67	0.33	3.33	48.33	1.0
97	PP1503-20-1-7	5.00	5.33	100	55.67	22.33	18.33	0.00	3.67	44.33	0.89
98	PP1503-22-1-5	6.00	6.00	100	43.00	22.00	30.33	1.00	3.67	57.00	1.0
99	PP1503-22-3-1	5.33	6.33	100	40.33	18.00	40.67	0.33	0.67	59.67	1.22
100	PP1503-22-3-3	5.67	6.00	100	47.67	20.00	29.67	0.00	2.67	52.33	1.56
101	PP1503-22-4-5	5.67	5.67	100	62.67	19.33	16.00	0.00	2.00	37.33	1.33
102	PP1503-25-4-4	6.33	6.00	100	41.33	22.33	36.00	0.00	0.33	58.67	1.22
103	PP1503-25-5-2	6.00	5.67	100	58.67	23.00	16.00	0.00	2.33	41.33	1.33
104	PP1503-25-5-6	6.33	6.33	100	31.33	22.67	44.33	0.00	1.67	68.67	1.0
105	PP1503-26-3-5	6.67	6.33	100	51.67	22.67	22.00	0.00	3.67	48.33	1.67
106	PP1503-27-2-8	5.67	6.00	100	52.00	21.00	25.00	0.33	1.67	48.00	1.08
107	PP1503-27-4-4	6.33	6.00	100	49.33	20.33	26.67	0.33	3.33	50.67	1.0

108	PP1503-28-1-3	6.67	6.00	100	45.67	22.00	31.00	0.00	1.33	54.33	1.0
109	PP1503-29-1-5	6.00	6.00	100	46.67	18.33	33.33	0.33	1.33	53.33	1.08
110	PP1503-29-2-1	6.00	6.67	100	21.67	24.33	50.67	0.00	3.33	78.33	0.92
111	PP1503-29-2-7	6.67	6.00	100	44.00	22.00	30.33	0.00	3.67	56.00	0.11
112	PP1503-3-1-3	6.33	7.00	100	47.00	21.67	28.67	0.00	2.67	53.00	1.44
113	PP1503-30-1-6	6.00	6.00	100	56.33	19.33	23.00	0.00	1.33	43.67	1.56
114	PP1503-5-2-4	6.33	5.67	100	43.67	20.67	32.00	0.00	3.67	56.33	1.0
115	PP1503-5-3-1	6.00	6.00	100	59.67	19.33	19.00	0.00	2.00	40.33	1.08
116	PP1503-5-3-3	5.67	6.00	100	63.00	23.33	10.33	0.33	3.00	37.00	0.56
117	PP1503-6-1-4	5.67	5.33	100	58.33	18.67	23.00	0.00	0.00	41.67	0.89
118	PP1503-6-2-5	6.00	5.33	100	43.33	24.67	25.67	0.67	5.67	56.67	1.33
119	PP1503-8-2-4	6.00	5.33	100	57.67	25.33	9.33	0.33	7.33	42.33	0.11
120	PP1503-8-3-2	6.00	6.67	100	42.33	22.00	32.33	0.33	3.00	57.67	0.77
121	PP1504-1-3-1	5.67	5.33	100	39.00	22.00	35.33	0.33	3.33	61.00	1.44
122	PP1504-2-1-3	6.33	7.00	100	36.67	19.33	40.67	0.00	3.33	63.33	1.17
123	PP1504-2-11-5	6.33	7.00	100	31.33	19.00	48.33	0.67	0.67	68.67	1.08
124	PP1504-2-2-1	5.67	5.67	100	44.67	23.67	27.33	0.00	4.33	55.33	1.22
125	PP1504-3-7-2	7.33	7.33	100	34.67	19.33	42.33	0.67	3.00	65.33	1.33
126	PP1504-4-8-2	6.67	7.00	100	23.00	25.67	47.33	0.33	3.67	77.00	1.56
127	PP1504-4-9-1	6.00	6.67	100	38.67	21.33	39.33	0.00	0.67	61.33	1.44
128	PP1504-4-9-2	5.67	6.67	100	26.33	19.67	53.33	0.33	0.33	73.67	1.11
129	PP1505-11-2-2	6.33	5.67	100	49.67	24.33	23.00	0.33	2.67	50.33	1.08
130	PP1505-11-2-4	6.67	6.00	100	42.67	19.00	37.00	0.33	1.00	57.33	1.0
131	PP1505-11-2-5	7.00	6.00	100	55.33	21.67	20.33	0.33	2.33	44.67	0.67
132	PP1505-11-2-6	6.33	5.33	100	35.67	18.00	42.33	2.67	1.33	64.33	0.33
133	PP1505-13-6-3	6.67	5.67	100	63.50	19.00	16.00	1.00	0.50	36.50	1.08
134	PP1505-2-3-1	6.67	5.33	100	29.33	23.67	46.33	0.00	0.67	70.67	1.22
135	PP1505-2-8-1	6.67	5.00	100	49.33	22.33	21.67	2.00	4.67	50.67	0.78
136	PP1505-20-5-2	6.67	6.33	100	58.00	16.67	23.00	2.00	0.33	42.00	1.08

137	PP1505-28-6-1	6.33	5.33	100	42.67	23.67	31.67	0.00	2.00	57.33	0.11
138	PP1505-29-4-1	5.67	6.00	100	28.67	24.67	44.67	0.00	2.00	71.33	1.0
139	PP1505-31-1-2	7.00	8.00	-	-	-	-	-	-	-	-
140	PP1505-32-3-2	6.33	5.67	100	41.33	19.33	38.33	0.00	1.00	58.67	1.08
141	PP1505-32-5-3	7.00	6.67	100	64.33	17.67	15.00	0.67	2.33	35.67	1.44
142	PP1505-34-3-6	6.33	5.67	100	55.33	19.67	22.33	0.00	2.67	44.67	0.44
143	PP1505-63-2-6	6.00	6.67	100	33.67	21.67	41.33	2.33	1.00	66.33	1.33
144	PP1505-36-4-1	6.67	4.67	100	45.67	20.67	31.67	1.33	0.67	54.33	0.67
145	PP1505-36-4-2	7.00	5.33	100	42.33	20.33	33.00	2.00	2.33	57.67	0.78
146	PP1505-36-4-4	6.67	6.00	100	46.67	22.67	27.67	0.33	2.67	53.33	0.89
147	PP1505-4-4-1	6.33	5.67	100	45.33	23.67	28.00	0.00	3.00	54.67	1.33
148	PP1505-59-4-1	6.33	5.67	100	47.00	19.00	31.00	0.33	2.67	53.00	0.99
149	PP1505-59-4-3	6.00	6.00	100	43.33	21.67	32.67	0.00	2.33	56.67	1.22
150	PP1505-59-4-4	6.33	6.33	100	44.67	21.67	31.00	0.00	2.67	55.33	0.89
151	PP1505-59-4-6	6.67	5.00	100	42.67	26.67	22.33	1.00	7.33	57.33	1.44
152	PP1505-59-7-1	7.00	6.00	100	27.67	22.00	46.67	1.00	2.67	72.33	1.89
153	PP1505-59-7-2	6.00	6.33	100	47.00	23.67	24.67	0.67	4.00	53.00	1.11
154	PP1505-61-4-1	6.67	7.00	100	51.33	20.67	24.00	0.33	3.67	48.67	1.42
155	PP1505-63-2-4	5.33	5.33	100	53.33	19.33	25.33	1.00	1.00	46.67	0.22
156	PP1505-63-2-5	6.33	6.67	100	33.50	24.00	37.50	1.00	4.00	66.50	1.89
158	ICP 8863	5.00	5.67	100	58.00	21.00	18.33	0.33	2.33	42.00	0.33
163	ICPL 87119	6.33	6.33	100	54.00	21.67	20.00	0.00	4.33	46.00	0.78
157	ENT 11	6.33	5.33	100	49.67	18.67	29.00	1.67	1.00	50.33	0.89
159	ICPL 332 WR	5.33	5.00	100	56.00	18.33	21.67	0.00	4.00	44.00	0.44
160	ICP 7035	5.67	5.33	100	60.67	19.00	17.00	0.33	3.00	39.33	0.44
161	ICPL 85010	7.33	7.67	100	48.00	21.00	28.67	0.00	2.33	52.00	0.89
162	ICPL 87	7.33	7.67	100	29.67	34.33	35.33	0.00	0.67	70.33	2.44
164	ICPL 88039	6.67	7.00	100	48.33	18.67	29.67	1.00	2.33	51.67	0.44
	**Fp**	<0.001	<0.001		<0.001	0.113	<0.001	0.010	0.192	<0.001	<0.001

	**Vr**	1.63	2.47		1.78	1.18	1.72	1.36	1.12	1.78	2.13
	**SE±**	0.40	0.40		7.11	1.82	6.97	0.43	1.38	7.10	1.066
	**LSD (P 0.05)**	1.10	1.00		19.77	5.06	19.39	1.20	3.85	19.77	0.3019
	**CV (%)**	11.20	10.40		25.70	15.10	42.30	216.10	101.30	23.60	0.8376

Note: Fp, *F* probability; Vr, variance; SE, standard error of the mean; LSD, least significant difference; CV, coefficient of variation.

Supplementary Table S6. Detached pod assay of the selected introgression lines for pod borer resistance under laboratory conditions during the 2019 rainy season at ICRISAT, Patancheru, India.

S. No.	Genotype identity	Pod damage rating	Pre-larval weight (mg)	Post-larval weight (mg)	Larval weight gain (%)
1	PP 1501 -14-5-3	5.00	13.2	56.00	333.10
2	PP1501-10-17-1	6.00	12.6	80.93	535.96
3	PP1501-10-17-2	4.33	15.1	99.73	559.65
4	PP1501-1-10-1	3.33	11.87	34.87	194.30
5	PP1501-1-10-4	6.00	10.63	77.03	620.53
6	PP1501-1-10-8	3.00	9.83	14.23	45.10
7	PP1501-1-12-3	5.67	13.03	75.57	490.75
8	PP1501-1-12-8	4.33	12.67	73.37	478.36
9	PP1501-1-13-2	5.33	11.7	89.60	646.29
10	PP1501-1-13-3	4.00	12.03	82.73	594.33
11	PP1501-1-13-4	3.33	11.17	56.40	438.56
12	PP1501-1-13-5	5.33	8.43	110.00	1203.33
13	PP1501-1-18-4	4.67	11.27	104.47	836.93
14	PP1501-1-20-2	4.33	12.63	46.47	257.84
15	PP1501-1-20-6	5.33	12.1	76.07	517.11
16	PP1501-12-1-1	3.00	9.6	30.70	221.83
17	PP1501-12-1-2	4.33	7.87	74.27	838.18
18	PP1501-12-1-3	3.00	7.87	15.57	97.76
19	PP1501-12-1-4	4.00	9.63	49.50	429.31
20	PP1501-12-1-5	3.33	9.3	61.57	539.79
21	PP1501-12-1-6	3.67	7.6	53.80	609.88
22	PP1501-12-1-7	5.67	6.83	71.37	940.78
23	PP1501-1-2-2	3.33	7.57	62.97	751.46
24	PP1501-1-22-1	3.67	9.2	51.27	456.57
25	PP1501-1-22-2	3.33	6.93	55.87	700.67
26	PP1501-1-22-3	3.00	7.23	32.60	390.51
27	PP1501-1-22-4	4.00	6.67	54.90	724.93
28	PP1501-1-22-5	4.67	6.93	53.43	659.88
29	PP1501-1-23-2	3.00	5.2	41.90	708.07
30	PP1501-1-23-3	3.67	8.5	64.17	693.76
31	PP1501-1-2-4	3.67	9.87	50.30	403.98
32	PP1501-14-1-5	3.67	9.63	51.03	438.71
33	PP1501-14-2-1	4.33	7.77	72.17	802.12
34	PP1501-14-2-3	3.67	7.27	20.03	175.62
35	PP1501-14-4-3	4.33	7.7	96.80	1126.60
36	PP1501-14-7-7	3.67	7.57	37.63	396.32
37	PP1501-1-5-2	3.33	10.03	49.03	414.93
38	PP1501-1-6-2	2.67	8.27	14.87	85.16
39	PP1501-16-6-5	3.33	7.97	49.93	525.54

40	PP1501-16-7-2	3.00	6.93	43.37	542.43
41	PP1501-16-7-6	3.00	7	53.73	736.85
42	PP1501-16-7-7	4.33	10.3	63.17	512.23
43	PP1501-1-8-2	2.67	9.73	60.27	502.50
44	PP1501-20-6-2	3.33	6.87	38.53	421.58
45	PP1501-2-13-2	2.33	5.27	18.40	251.43
46	PP1501-2-16-4	4.00	5.37	49.80	814.88
47	PP1501-2-2-5	4.67	11.33	65.33	462.67
48	PP1501-2-3-6	4.33	8.17	67.03	720.14
49	PP1501-2-4-1	3.00	9.07	46.40	405.56
50	PP1501-2-5-1	5.67	8.83	66.13	650.16
51	PP1501-2-6-2	4.00	7.37	55.43	651.78
52	PP1501-2-9-4	3.67	8.5	50.77	513.96
53	PP1501-3-11-2	5.33	8.67	66.77	663.24
54	PP1501-3-15-1	4.67	9.17	65.70	628.01
55	PP1501-3-17-3	2.67	7.87	52.97	577.79
56	PP1501-4-12-1	3.33	7.27	51.77	623.54
57	PP1501-4-12-2	3.00	7.5	29.03	289.57
58	PP1501-4-12-4	4.33	6.57	40.13	485.35
59	PP1501-4-12-5	3.33	6.5	52.87	710.85
60	PP1501-4-14-3	4.67	8.97	52.83	488.22
61	PP1501-4-14-4	3.67	7.87	13.00	65.54
62	PP1501-4-17-1	3.00	7.4	31.40	327.66
63	PP1501-4-17-2	3.00	7.37	47.20	522.19
64	PP1501-4-17-3	4.67	7.2	63.83	786.72
65	PP1501-4-17-7	3.33	6.57	29.37	359.15
66	PP1501-4-20-1	4.00	5.93	34.07	496.44
67	PP1501-4-21-6	2.33	6.17	48.90	706.50
68	PP1501-4-8-2	2.67	5.67	37.10	550.77
69	PP1501-4-9-2	2.67	6.73	45.73	588.33
70	PP1501-4-9-3	3.33	6.13	60.33	878.26
71	PP1501-5-14-1	3.67	6.7	12.60	88.70
72	PP1501-5-14-3	3.00	6.3	14.83	137.28
73	PP1501-5-14-4	3.00	5.93	11.73	96.72
74	PP1501-5-15-3	3.33	6	37.90	530.26
75	PP1501-5-15-7	5.00	5.4	35.00	552.91
76	PP1501-6-12-2	6.50	13.3	88.00	559.21
77	PP1501-7-19-5	4.50	5.7	46.45	716.79
78	PP1501-9-18-1	5.00	12.7	38.80	206.46
79	PP1501-9-9-2	6.50	13.75	70.47	406.50
80	PP1503-10-5-4	7.00	16.15	67.60	318.31
81	PP1503-10-5-6	6.50	12.85	30.03	151.90
82	PP1503-11-1-1	6.50	13.35	83.85	511.79
83	PP1503-11-1-4	5.50	16.6	45.47	172.24
84	PP1503-12-1-7	5.00	17.1	41.80	149.23

85	PP1503-12-2-2	4.50	10.3	34.52	237.53
86	PP1503-12-2-6	6.00	17.35	88.65	410.57
87	PP1503-14-2-7	6.50	7.5	58.35	405.48
88	PP1503-15-1-2	6.00	15.2	51.10	238.14
89	PP1503-15-1-7	5.50	11.95	47.40	293.83
90	PP1503-15-2-2	5.00	14.35	26.68	85.88
91	PP1503-15-3-3	6.50	16.8	72.70	329.80
92	PP1503-15-3-4	6.50	14.9	83.30	458.30
93	PP1503-16-3-8	6.50	12.9	73.00	459.66
94	PP1503-18-1-7	6.50	16.55	76.30	352.60
95	PP1503-20-1-2	7.50	6.73	70.95	299.71
96	PP1503-20-1-5	7.50	17.15	97.95	470.71
97	PP1503-20-1-7	7.00	15.15	51.15	241.46
98	PP1503-22-1-5	7.50	11.95	76.70	544.70
99	PP1503-22-3-1	6.50	10.85	43.40	302.94
100	PP1503-22-3-3	7.00	15.05	55.60	277.04
101	PP1503-22-4-5	4.00	12.63	19.43	52.38
102	PP1503-25-4-4	5.50	10.85	44.80	323.61
103	PP1503-25-5-2	5.00	13.15	59.10	349.15
104	PP1503-25-5-6	6.00	13.85	35.30	145.24
105	PP1503-26-3-5	7.00	13.85	87.55	540.57
106	PP1503-27-2-8	6.00	12.8	43.95	219.93
107	PP1503-27-4-4	6.00	19.25	81.55	320.24
108	PP1503-28-1-3	6.50	19.55	75.40	283.51
109	PP1503-29-1-5	7.00	14	67.80	381.40
110	PP1503-29-2-1	5.50	13.2	44.80	239.38
111	PP1503-29-2-7	8.00	15.7	84.00	437.00
112	PP1503-30-1-6	7.50	17.75	66.45	273.97
113	PP1503-3-1-3	3.00	9.75	11.38	16.76
114	PP1503-5-2-4	6.00	16.15	69.95	330.38
115	PP1503-5-3-1	5.50	12.3	94.15	660.28
116	PP1503-5-3-3	4.50	12.9	40.67	213.06
117	PP1503-6-1-4	5.00	14.8	38.87	163.51
118	PP1503-6-2-5	5.50	10.45	35.07	232.30
119	PP1503-8-2-4	5.00	12.95	60.05	354.88
120	PP1503-8-3-2	7.00	17.55	59.65	242.75
121	PP1504-1-3-1	2.33	13.2	14.43	114.88
122	PP1504-2-11-5	2.67	18.5	13.57	69.73
123	PP1504-2-1-3	4.33	14.65	57.03	658.40
124	PP1504-2-2-1	5.67	18.7	70.00	690.50
125	PP1504-3-7-2	4.67	14.3	24.57	208.95
126	PP1504-4-8-2	5.00	17.85	38.43	451.60
127	PP1504-4-9-1	4.00	22.3	71.10	792.73
128	PP1504-4-9-2	4.00	20.8	18.30	103.41
129	PP1505-11-2-2	7.00	16.45	153.93	844.50

130	PP1505-11-2-4	6.50	20.75	125.10	501.93
131	PP1505-11-2-5	5.00	17.4	56.25	230.46
132	PP1505-11-2-6	6.00	18.25	79.72	330.03
133	PP1505-13-6-3	6.50	17.1	80.03	367.86
134	PP1505-20-5-2	6.50	21.35	92.55	344.69
135	PP1505-2-3-1	8.50	8.8	81.50	828.24
136	PP1505-2-8-1	7.50	20.35	113.80	459.44
137	PP1505-28-6-1	4.00	12.47	99.00	723.27
138	PP1505-29-4-1	5.67	13.47	89.27	536.10
139	PP1505-31-1-2	4.71	11.33	60.00	477.00
140	PP1505-32-3-2	4.33	11.57	102.30	816.44
141	PP1505-32-5-3	3.33	11.17	116.03	943.59
142	PP1505-34-3-6	4.00	7.5	44.20	349.21
143	PP1505-63-2-6	4.33	7	90.07	883.02
144	PP1505-36-4-1	4.33	7.93	52.60	340.40
145	PP1505-36-4-2	5.00	10.4	41.87	300.04
146	PP1505-36-4-4	4.67	10.07	113.13	1031.08
147	PP1505-4-4-1	3.67	10.3	77.07	660.47
148	PP1505-59-4-1	4.33	7.93	72.80	783.73
149	PP1505-59-4-3	6.50	10.30	73.33	352.83
150	PP1505-59-4-4	5.00	10.7	105.27	901.95
151	PP1505-59-4-6	3.00	7.9	60.83	661.00
152	PP1505-59-7-1	4.67	7.63	50.03	559.81
153	PP1505-59-7-2	6.50	10.30	79.47	459.30
154	PP1505-61-4-1	3.67	9.6	68.53	600.56
155	PP1505-63-2-4	6.00	7.77	87.20	1010.33
156	PP1505-63-2-5	4.00	9	85.17	884.10
**Recurrent parents**					
157	ICP 8863	5.00	11.37	85.93	653.56
158	ICPL 87119	3.33	13.73	65.33	370.82
**Checks**					
159	ENT 11	5.67	13.17	93.17	628.48
160	ICPL 332 WR	5.67	11.90	113.63	853.99
161	ICPL 88039	4.33	12.97	75.60	490.47
162	ICP 7035	3.33	14.60	56.63	287.43
163	ICPL 85010	4.67	10.60	80.00	651.73
164	ICPL 87	3.00	13.03	64.40	420.32
	**Fp**	**<0.001**	**<0.001**	**<0.001**	**<0.001**
	**Mean**	**4.70**	**11.30**	**60.00**	**477.00**
	**SE ±**	**0.39**	**0.95**	**9.89**	**88.02**
	**LSD (P 0.05)**	**1.09**	**2.65**	**27.50**	**244.89**
	**CV (%)**	**14.40**	**14.60**	**28.50**	**32.00**

Note: Fp, *F* probability; Vr, variance; SE, standard error of the mean; LSD, least significant difference; CV, coefficient of variation.

Supplementary Table S7. Evaluation of introgression lines for pod borer damage and important agronomic traits during the 2019 rainy season at Warangal, India.

S.No.	Line/Phenotype	Days to first flowering	Days to 50% flowering	Days to maturity	Recovery resistance score	Total No. of pods per 5 plants	Pod borer damage per 5 plants (%)	Spotted borer damage per 5 plants (%)	Pod fly damage per 5 plants (%)	Pod wasp damage per 5 plants (%)	Plume moth damage per 5 plants (%)	Total pod borer complex damage (%)	Total healthy pods (%)
1	ENT 11	100.7	108.7	154.7	5.7	393.3	8.1	8.9	13.1	0.0	0.0	30.1	69.9
2	ICP 7035	101.3	109.3	153.3	6.0	207.7	7.4	13.9	12.4	0.0	0.0	33.7	66.3
3	ICP 8863	97.0	105.0	145.0	8.0	610.3	9.9	5.4	14.3	0.0	0.0	29.5	70.5
4	ICPL 332 WR	98.3	106.3	146.3	5.7	777.7	4.4	12.1	21.1	0.0	0.0	37.6	62.4
5	ICPL 85010	76.3	84.3	128.3	4.7	224.0	14.7	12.4	17.5	0.0	0.0	44.7	55.3
6	ICPL 87	72.7	80.7	122.0	7.3	401.7	17.6	5.4	5.6	0.0	0.0	28.7	71.3
7	ICPL 87119	102.0	110.0	161.3	7.0	843.3	6.3	9.0	19.0	0.0	0.0	34.3	65.7
8	ICPL 88039	79.7	87.7	127.7	6.7	419.0	20.0	3.8	17.0	0.0	0.0	40.8	59.2
9	PP 1501 -14-5-3	96.7	104.7	146.3	7.0	593.3	11.8	7.7	9.1	0.0	0.0	28.6	71.4
10	PP1501-1-10-1	101.3	109.3	151.3	7.0	807.7	16.2	13.7	16.7	0.0	0.0	46.6	53.4
11	PP1501-1-10-4	116.0	124.0	166.0	6.3	571.7	5.0	7.7	17.7	0.0	0.0	30.4	69.6
12	PP1501-1-10-8	107.0	115.0	158.3	6.0	768.3	10.4	7.8	18.6	0.0	0.0	36.8	63.2
13	PP1501-1-12-3	99.0	107.0	151.0	6.7	700.7	4.9	5.4	25.0	0.0	0.0	35.4	64.6
14	PP1501-1-12-8	97.7	105.7	147.0	7.0	717.7	17.0	10.0	19.7	0.0	0.0	46.7	53.3
15	PP1501-1-13-2	108.0	116.0	156.0	7.0	466.3	8.5	6.1	18.1	0.0	0.0	32.8	67.2
16	PP1501-1-13-3	104.3	112.3	153.3	6.0	344.0	10.6	8.1	7.7	0.0	0.0	26.4	73.6
17	PP1501-1-13-4	98.0	106.0	144.0	7.0	713.0	8.8	8.4	12.2	0.0	0.0	29.4	70.6
18	PP1501-1-13-5	85.7	93.7	134.7	7.0	540.3	16.0	5.4	10.6	0.0	0.0	32.0	68.0
19	PP1501-1-18-4	105.7	113.7	156.0	7.0	584.7	5.8	8.3	17.1	0.0	0.0	31.2	68.8
20	PP1501-1-2-2	97.3	105.3	146.7	7.0	518.0	12.9	7.2	17.8	0.0	0.0	37.8	62.2

21	PP1501-1-2-4	103.3	111.3	147.7	6.3	703.0	11.3	15.5	16.7	0.1	0.0	43.6	56.4
22	PP1501-1-20-2	103.7	111.7	153.3	7.3	485.7	11.8	12.2	13.4	0.0	0.0	37.4	62.6
23	PP1501-1-20-6	111.0	119.0	163.0	6.7	673.7	9.6	9.9	27.2	0.0	0.0	46.7	53.3
24	PP1501-1-22-1	102.0	110.0	153.3	7.3	224.3	19.3	7.7	12.9	0.0	0.0	40.0	60.0
25	PP1501-1-22-2	89.0	97.0	140.0	7.7	512.3	10.2	4.2	6.9	1.1	0.0	22.3	77.7
26	PP1501-1-22-3	96.3	104.3	144.3	7.0	672.7	14.6	6.3	9.2	0.0	0.0	30.1	69.9
27	PP1501-1-22-4	91.0	99.0	140.0	7.0	514.0	24.1	9.9	9.1	0.0	0.0	43.1	56.9
28	PP1501-1-22-5	92.0	100.0	142.0	6.7	670.7	10.0	6.5	7.2	0.0	0.0	23.7	76.3
29	PP1501-1-23-2	99.3	107.3	148.0	5.3	523.7	7.8	6.2	9.2	0.0	0.0	23.2	76.8
30	PP1501-1-23-3	101.0	109.0	149.0	7.0	218.3	17.3	9.7	17.4	0.0	0.0	44.5	55.5
31	PP1501-1-5-2	99.3	107.3	146.3	7.0	606.3	16.1	7.0	6.2	0.0	0.0	29.2	70.8
32	PP1501-1-6-2	91.0	99.0	140.0	7.0	506.7	13.4	3.8	19.8	0.0	0.0	37.1	62.9
33	PP1501-1-8-2	113.3	121.3	161.0	6.7	788.3	13.0	5.8	23.7	0.0	0.0	42.4	57.6
34	PP1501-10-17-1	98.0	106.0	151.3	7.0	328.0	11.2	12.6	16.5	0.0	0.0	40.3	59.7
35	PP1501-10-17-2	95.7	103.7	144.7	7.0	318.3	22.8	7.1	24.5	0.0	0.0	54.3	45.7
36	PP1501-12-1-1	126.0	134.0	176.7	5.7	691.7	12.4	13.1	7.8	0.0	0.0	33.3	66.7
37	PP1501-12-1-2	126.3	134.3	179.7	7.3	446.0	7.5	2.4	21.8	0.0	0.0	31.7	68.3
38	PP1501-12-1-3	115.0	123.0	164.7	6.3	700.3	8.3	7.4	17.8	0.0	0.0	33.4	66.6
39	PP1501-12-1-4	108.7	116.7	159.3	5.3	799.7	13.6	17.1	14.3	0.0	0.0	45.0	55.0
40	PP1501-12-1-5	109.7	117.7	157.0	5.3	1140.7	10.6	15.8	8.7	0.0	0.0	35.1	64.9
41	PP1501-12-1-6	127.7	135.7	176.3	6.0	1042.7	9.3	4.7	11.8	0.0	0.0	25.8	74.2
42	PP1501-12-1-7	121.3	129.3	171.7	6.3	769.0	11.9	10.7	17.6	0.0	0.0	40.3	59.7
43	PP1501-14-1-5	107.7	115.7	158.0	7.0	584.7	7.5	14.6	5.8	0.0	0.0	27.9	72.1
44	PP1501-14-2-1	125.3	133.3	177.7	5.7	733.3	7.0	6.9	19.8	0.0	0.0	33.7	66.3
45	PP1501-14-2-3	110.7	118.7	161.3	6.3	764.7	7.6	7.1	9.6	0.0	0.0	24.3	75.7
46	PP1501-14-4-3	99.7	107.7	150.0	6.3	675.0	14.7	11.2	16.3	0.0	0.0	42.1	57.9
47	PP1501-14-7-7	123.3	131.3	172.0	6.7	751.3	10.4	10.1	13.1	0.0	0.0	33.6	66.4
48	PP1501-16-6-5	121.7	129.7	170.3	6.0	566.0	12.7	13.7	16.3	0.0	0.0	42.7	57.3
49	PP1501-16-7-2	131.0	139.0	180.3	6.3	693.3	3.8	8.0	8.4	0.0	0.0	20.2	79.8

50	PP1501-16-7-6	128.0	136.0	178.3	4.3	585.0	7.0	10.4	8.6	0.0	0.0	26.0	74.0
51	PP1501-16-7-7	124.3	132.3	173.3	6.3	632.3	7.0	6.4	12.3	0.0	0.0	25.7	74.3
52	PP1501-2-13-2	100.0	108.0	148.0	7.3	643.7	6.8	5.7	14.3	0.0	0.0	26.8	73.2
53	PP1501-2-16-4	112.0	120.0	160.7	6.7	796.0	8.3	3.3	4.1	0.0	0.0	15.7	84.3
54	PP1501-2-3-6	90.7	98.7	141.0	6.3	505.7	20.6	8.2	9.4	0.0	0.0	38.3	61.7
55	PP1501-2-4-1	102.0	110.0	150.0	6,33	672.7	14.4	10.9	10.4	0.0	0.0	35.7	64.3
56	PP1501-2-5-1	101.0	109.0	154.3	6.7	546.3	6.7	4.1	9.6	0.0	0.0	20.4	79.6
57	PP1501-2-6-2	96.7	104.7	146.7	7.7	438.7	9.8	8.6	16.7	0.0	0.0	35.0	65.0
58	PP1501-2-9-4	108.7	116.7	161.3	7.0	496.3	17.6	7.4	5.5	0.0	0.0	30.4	69.6
59	PP1501-20-6-2	122.0	130.0	172.7	6.3	383.0	9.8	9.7	32.1	0.0	0.0	51.6	48.4
60	PP1501-3-11-2	100.0	108.0	150.0	7.0	419.0	12.1	6.2	6.3	0.0	0.0	24.6	75.4
61	PP1501-3-15-1	113.3	121.3	166.3	7.0	873.0	15.7	10.9	20.2	0.0	0.0	46.8	53.2
62	PP1501-3-17-3	103.0	111.0	154.0	7.3	529.0	10.4	6.3	19.9	0.0	0.0	36.6	63.4
63	PP1501-4-12-1	84.0	92.0	141.3	6.7	460.0	8.4	7.2	10.0	0.0	0.0	25.5	74.5
64	PP1501-4-12-2	96.0	104.0	144.0	6.3	657.3	22.0	6.0	6.1	0.0	0.0	34.1	65.9
65	PP1501-4-12-4	88.0	96.0	142.0	7.0	471.0	17.6	12.2	11.5	0.0	0.0	41.3	58.7
66	PP1501-4-12-5	100.0	108.0	151.0	7.7	537.0	16.5	10.7	7.6	0.0	0.0	34.8	65.2
67	PP1501-4-14-3	100.0	108.0	150.0	7.0	360.0	13.2	9.0	12.8	0.3	0.0	35.2	64.8
68	PP1501-4-14-4	108.3	116.3	146.7	6.3	498.3	11.9	5.2	22.5	0.0	0.0	39.6	60.4
69	PP1501-4-17-1	126.3	134.3	173.3	7.0	414.3	6.3	9.4	27.3	0.0	0.0	42.9	57.1
70	PP1501-4-17-2	111.0	119.0	163.3	7.0	995.7	16.5	9.1	9.5	0.0	0.0	35.2	64.8
71	PP1501-4-17-3	98.3	106.3	148.7	4.3	562.3	6.5	7.0	11.1	0.0	0.0	24.6	75.4
72	PP1501-4-17-7	89.7	97.7	141.0	5.7	500.3	15.1	11.0	9.7	0.0	0.0	35.9	64.1
73	PP1501-4-20-1	104.0	112.0	152.7	7.3	431.7	7.6	11.3	13.8	0.0	0.0	32.7	67.3
74	PP1501-4-21-6	106.7	114.7	155.3	4.7	772.3	7.6	6.4	10.0	0.0	0.0	24.0	76.0
75	PP1501-4-8-2	107.3	115.3	156.0	6.0	554.3	11.9	10.3	11.6	0.0	0.0	33.8	66.2
76	PP1501-4-9-2	106.0	114.0	156.0	6.3	589.7	9.9	10.8	8.3	0.0	0.0	29.0	71.0
77	PP1501-4-9-3	102.7	110.7	154.0	7.3	664.7	20.7	6.2	6.7	0.0	0.0	33.5	66.5
78	PP1501-5-14-1	107.0	115.0	156.0	7.0	518.7	9.9	3.5	10.5	0.0	0.0	24.0	76.0

79	PP1501-5-14-3	110.3	118.3	158.0	7.0	821.0	16.0	8.9	18.8	0.0	0.0	43.7	56.3
80	PP1501-5-14-4	118.3	126.3	168.0	5.7	721.0	9.5	9.4	24.3	0.0	0.0	43.3	56.7
81	PP1501-5-15-3	101.7	109.7	149.0	6.7	795.7	10.3	11.8	20.2	0.0	0.0	42.3	57.7
82	PP1501-5-15-7	110.0	118.0	160.0	7.3	637.7	10.4	10.3	9.2	0.0	0.0	30.0	70.0
83	PP1501-6-12-2	90.0	98.0	141.0	7.0	517.7	17.3	10.1	10.2	0.0	0.0	37.5	62.5
84	PP1501-7-19-5	96.3	104.3	144.0	7.3	789.7	12.3	7.0	17.5	0.0	0.0	36.8	63.2
85	PP1501-9-18-1	102.0	110.0	152.0	6.3	775.0	5.0	8.3	28.3	0.0	0.0	41.5	58.5
86	PP1501-9-9-2	113.3	121.3	162.0	6.3	579.3	14.7	8.8	19.0	0.0	0.0	42.4	57.6
87	PP1503-10-5-4	125.7	133.7	177.7	6.0	1019.7	10.0	13.1	18.1	0.0	0.0	41.3	58.7
88	PP1503-10-5-6	121.3	129.3	174.0	6.0	654.7	15.9	10.2	11.6	0.0	0.0	37.7	62.3
89	PP1503-12-1-7	114.3	122.3	164.3	6.7	565.0	12.3	15.6	19.7	0.0	0.0	47.6	52.4
90	PP1503-12-2-2	128.3	136.3	178.7	6.3	804.7	10.4	7.7	14.5	0.0	0.0	32.6	67.4
91	PP1503-12-2-6	103.0	111.0	152.0	6.7	1064.3	14.0	8.1	9.7	0.0	0.0	31.8	68.2
92	PP1503-14-2-7	126.7	134.7	176.3	6.7	793.7	6.3	7.4	21.2	0.0	0.0	34.9	65.1
93	PP1503-15-1-2	107.0	115.0	159.3	4.7	617.3	16.2	7.8	10.8	0.0	0.0	34.7	65.3
94	PP1503-15-1-7	117.0	125.0	164.0	6.3	985.0	6.8	4.0	2.8	0.0	0.0	13.7	86.3
95	PP1503-15-2-2	124.0	132.0	173.3	7.3	807.3	20.2	15.1	22.7	0.0	0.0	58.0	42.0
96	PP1503-15-3-3	103.7	111.7	153.3	6.3	750.3	18.1	21.7	13.3	0.0	0.0	53.1	46.9
97	PP1503-15-3-4	99.0	107.0	151.0	6.7	786.3	7.2	10.0	11.4	0.0	0.0	28.6	71.4
98	PP1503-16-3-8	100.3	108.3	149.7	6.7	449.3	11.9	6.4	16.5	0.0	0.0	34.9	65.1
99	PP1503-20-1-2	108.0	116.0	160.0	6.7	729.7	12.0	11.6	16.0	0.0	0.0	39.6	60.4
100	PP1503-20-1-5	106.0	114.0	156.3	5.7	807.3	13.4	16.3	11.8	0.0	0.0	41.5	58.5
101	PP1503-20-1-7	105.7	113.7	152.7	7.0	737.3	10.0	8.7	9.5	0.0	0.0	28.2	71.8
102	PP1503-22-1-5	101.7	109.7	158.0	7.7	624.0	5.8	13.3	19.8	0.0	0.0	38.9	61.1
103	PP1503-22-3-3	120.3	128.3	169.0	6.7	688.3	9.5	9.0	17.2	0.0	0.0	35.7	64.3
104	PP1503-22-4-5	120.0	128.0	170.0	5.7	906.7	15.5	12.7	14.3	0.0	0.0	42.4	57.6
105	PP1503-25-4-4	108.0	116.0	159.3	5.0	841.0	15.8	15.4	13.5	0.0	0.0	44.7	55.3
106	PP1503-25-5-2	117.7	125.7	170.0	6.7	761.3	10.3	6.0	12.8	0.0	0.0	29.1	70.9
107	PP1503-26-3-5	96.0	104.0	144.0	6.0	770.7	4.1	4.2	19.2	0.0	0.0	27.5	72.5

108	PP1503-27-2-8	102.3	110.3	152.7	5.7	739.3	6.0	8.6	18.7	0.0	0.0	33.3	66.7
109	PP1503-27-4-4	108.7	116.7	164.7	5.7	1168.3	12.5	8.0	14.8	0.0	0.0	35.3	64.7
110	PP1503-28-1-3	105.7	113.7	157.7	6.3	1007.0	12.9	13.6	11.2	0.0	0.0	37.8	62.2
111	PP1503-29-1-5	116.7	124.7	168.0	6.7	702.0	24.8	12.0	8.7	0.0	0.0	45.5	54.5
112	PP1503-29-2-7	125.7	133.7	176.3	6.7	1245.3	9.1	3.8	21.6	0.0	0.0	34.5	65.5
113	PP1503-30-1-6	120.0	128.0	170.0	6.7	1016.0	6.1	4.7	12.4	0.0	0.0	23.1	76.9
114	PP1503-5-2-4	100.7	108.7	150.0	6.3	776.3	13.2	6.5	16.6	0.0	0.0	36.3	63.7
115	PP1503-5-3-1	122.3	130.3	172.0	6.7	503.0	12.3	10.0	21.4	0.0	0.0	43.8	56.2
116	PP1503-5-3-3	111.0	119.0	159.3	6.0	528.0	12.5	10.8	23.3	0.0	0.0	46.7	53.3
117	PP1503-6-1-4	128.7	136.7	174.0	6.7	776.0	5.2	8.4	17.9	0.0	0.0	31.5	68.5
118	PP1503-6-2-5	104.7	112.7	156.0	6.0	727.3	15.6	12.9	15.8	0.0	0.0	44.3	55.7
119	PP1503-8-2-4	120.7	128.7	170.7	4.3	404.3	10.1	8.3	6.6	0.0	0.0	25.0	75.0
120	PP1503-8-3-2	102.7	110.7	148.0	6.3	884.0	11.7	8.5	13.1	0.0	0.0	33.3	66.7
121	PP1504-1-3-1	98.7	106.7	145.3	5.0	942.3	12.7	6.3	14.9	0.0	0.0	33.9	66.1
122	PP1504-2-2-1	108.3	116.3	157.3	7.0	654.3	8.0	7.7	23.9	0.0	0.0	39.6	60.4
123	PP1505-11-2-2	125.0	133.0	176.3	7.0	281.7	6.5	11.2	5.8	0.0	0.0	23.5	76.5
124	PP1505-11-2-4	109.0	117.0	157.0	7.0	805.0	14.7	9.9	6.5	0.0	0.0	31.1	68.9
125	PP1505-11-2-5	101.0	109.0	150.3	7.0	495.3	11.8	10.1	11.0	0.0	0.0	32.9	67.1
126	PP1505-11-2-6	102.7	110.7	152.7	6.7	531.7	6.2	8.7	11.4	0.0	0.0	26.3	73.7
127	PP1505-13-6-3	101.3	109.3	152.3	6.7	446.3	14.1	8.2	6.9	0.0	0.0	29.1	70.9
128	PP1505-2-3-1	109.3	117.3	160.7	7.0	724.3	12.1	12.5	12.5	0.0	0.0	37.1	62.9
129	PP1505-2-8-1	88.7	96.7	140.3	6.7	820.7	6.5	2.9	18.4	0.0	0.0	27.8	72.2
130	PP1505-20-5-2	117.0	125.0	173.3	7.0	573.0	11.8	13.5	12.6	0.0	0.0	37.8	62.2
131	PP1505-28-6-1	99.7	107.7	149.0	6.7	226.7	7.2	6.5	16.5	0.0	0.0	30.2	69.8
132	PP1505-32-3-2	100.0	108.0	148.3	5.7	1066.3	13.6	8.2	16.9	0.0	0.0	38.7	61.3
133	PP1505-32-5-3	107.0	115.0	156.7	7.3	324.3	8.9	7.9	9.3	0.0	0.0	26.1	73.9
134	PP1505-34-3-6	127.3	135.3	180.3	6.3	560.0	16.1	11.9	14.1	0.0	0.0	42.1	57.9
135	PP1505-36-4-1	131.3	139.3	180.3	6.0	397.3	8.8	11.4	13.0	0.0	0.0	33.2	66.8
136	PP1505-36-4-2	108.3	116.3	161.0	6.0	1095.0	6.4	8.9	11.7	0.0	0.0	26.9	73.1

137	PP1505-36-4-4	111.0	119.0	160.7	4.7	744.0	12.5	6.2	8.0	0.0	0.0	26.7	73.3
138	PP1505-4-4-1	100.7	108.7	146.0	6.3	587.7	9.8	28.8	11.6	0.7	0.0	50.9	49.1
139	PP1505-59-4-1	128.3	136.3	181.0	5.3	1091.3	5.3	5.3	11.8	0.0	0.0	22.4	77.6
140	PP1505-59-4-3	131.3	139.3	180.0	5.0	1038.7	12.2	8.6	12.1	0.0	0.0	32.9	67.1
141	PP1505-59-4-4	119.7	127.7	170.0	7.0	871.7	6.3	11.5	19.5	0.0	0.0	37.3	62.7
142	PP1505-59-4-6	104.0	112.0	152.0	6.0	959.3	6.5	5.8	21.7	0.0	0.0	34.0	66.0
143	PP1505-59-7-1	121.0	129.0	172.7	7.0	790.3	10.1	8.4	5.2	0.0	0.0	23.7	76.3
144	PP1505-59-7-2	118.0	126.0	169.7	6.7	590.0	13.1	15.1	23.6	0.0	0.0	51.8	48.2
	**Fp**	**<0.001**	**<0.001**	**<0.001**	**0.106**	**0.003**	**<0.001**	**0.346**	**0.088**	**0.528**	*****	**0.015**	**0.015**
	**Vr**	**139.17**	**139.17**	**112.99**	**1.19**	**1.47**	**1.71**	**1.06**	**1.21**	**0.99**	*****	**1.36**	**1.36**
	**Mean**	**107.33**	**115.33**	**157.33**	**6.47**	**658.00**	**11.45**	**9.15**	**14.25**	**0.02**	**0.00**	**34.87**	**65.13**
	**SE ±**	**1.02**	**1.02**	**1.16**	**0.6735**	**174.60**	**3.36**	**3.59**	**5.17**	**0.11**	**0.00**	**6.87**	**6.87**
	**LSD (P 0.05)**	**2.83**	**2.83**	**3.22**	**1.8747**	**486.00**	**9.36**	**10.01**	**14.40**	**0.30**	*****	**19.13**	**19.13**
	**CV (%)**	**1.60**	**1.50**	**1.30**	**18**	**46.00**	**50.80**	**68.00**	**62.90**	**125.20**	**0.00**	**34.10**	**18.30**

Note: Fp, *F* probability; Vr, variance; SE, standard error of the mean; LSD, least significant difference; CV, coefficient of variation.

Supplementary Table S8. Trichome density (mm²) in the 39 most promising pod borer resistant introgression lines, as well as parents and checks, during the 2020 rainy season at ICRISAT, Patancheru, India.

S.No.	Genotype	Trichome density (mm²) on adaxial leaf surface				Trichome density (mm²) on abaxial leaf surface			
		Type A	Type B	Type C	Type D	Type A	Type B	Type C	Type D
1	ENT 11	1.00	34.33	23.67	3.33	3.00	16.33	36.00	6.00
2	ICP 8863	0.67	16.00	35.00	3.67	0.00	12.67	50.33	7.67
3	ICPL 332 WR	2.33	29.67	33.00	2.33	1.33	17.67	51.67	4.67
4	ICP 7035	1.00	30.00	29.00	3.00	0.00	11.67	58.33	6.67
5	ICPL 85010	0.33	20.33	18.00	2.33	1.67	16.67	26.67	4.33
6	ICPL 87	0.67	30.00	14.67	1.67	0.00	14.33	46.00	5.33
7	ICPL 87119	1.33	57.67	14.67	3.33	2.33	27.00	49.67	5.33
8	ICPL 88039	1.67	24.33	22.33	3.33	2.00	10.33	51.33	8.00
9	PP1501-1-10-1	1.33	33.67	9.67	1.33	2.33	17.00	27.33	4.33
10	PP1501-1-10-8	0.33	18.33	44.33	5.00	0.00	12.67	45.33	6.00
11	PP1501-1-13-5	0.33	24.00	27.00	2.67	2.00	25.67	53.33	6.67
12	PP1501-1-18-4	2.67	39.33	23.00	3.67	5.00	18.67	41.67	6.00
13	PP1501-1-22-3	1.67	17.33	31.67	3.33	1.00	14.00	62.67	4.33
14	PP1501-1-23-2	0.67	25.67	17.33	2.67	1.00	18.00	36.00	7.33
15	PP1501-1-23-3	0.33	31.00	22.67	2.67	2.00	11.00	39.67	4.67
16	PP1501-1-6-2	1.00	28.67	36.00	3.00	1.33	14.67	46.33	6.33
17	PP1501-10-17-2	0.67	29.33	28.00	4.00	3.00	15.33	58.67	6.33
18	PP1501-12-1-2	2.33	32.67	27.33	2.33	2.67	20.00	32.33	4.00
19	PP1501-12-1-5	1.00	40.00	30.67	2.67	3.33	18.00	56.00	9.00
20	PP1501-12-1-6	0.67	35.67	28.33	4.00	1.33	16.67	55.00	3.67
21	PP1501-14-1-5	0.00	39.33	30.67	3.00	1.33	12.67	60.33	6.33
22	PP1501-14-7-7	2.00	46.33	10.33	3.33	1.33	25.67	48.00	8.00
23	PP1501-16-7-2	0.33	28.67	37.67	5.67	1.67	19.33	53.67	8.67
24	PP1501-2-3-6	1.00	28.67	32.33	3.33	2.00	19.33	48.00	5.33
25	PP1501-2-9-4	0.67	21.00	18.67	3.33	1.67	19.67	62.33	4.33
26	PP1501-20-6-2	0.00	24.67	46.67	4.67	1.33	26.00	62.67	5.33
27	PP1501-3-17-3	2.00	33.67	30.33	1.67	1.00	16.00	44.33	6.33
28	PP1501-4-17-3	1.33	28.67	15.67	1.00	0.00	12.67	32.33	4.67
29	PP1501-4-17-7	2.33	20.33	18.00	2.00	2.67	13.33	36.00	4.00
30	PP1501-4-21-6	0.67	26.67	28.67	3.67	1.33	13.33	47.00	6.00
31	PP1503-20-1-7	1.33	20.00	45.67	5.33	1.00	13.67	64.33	12.33
32	PP1503-25-5-2	0.00	32.00	28.33	4.00	2.67	16.33	67.33	7.67
33	PP1503-5-2-4	1.67	49.67	35.00	3.00	1.00	19.00	58.33	5.33
34	PP1503-5-3-1	1.33	34.00	27.67	3.00	1.00	24.00	53.00	9.33
35	PP1503-5-3-3	0.33	46.00	38.67	2.33	5.00	26.67	51.67	6.00
36	PP1503-6-1-4	0.00	25.33	36.00	4.00	0.67	10.33	52.33	9.67
37	PP1505-11-2-4	1.33	15.33	24.00	3.00	1.33	13.67	48.33	5.67
38	PP1505-11-2-5	2.33	24.00	25.33	5.67	1.67	18.00	59.33	7.33
39	PP1505-11-2-6	0.67	22.00	39.67	5.67	0.33	23.33	54.67	5.33
40	PP1505-13-6-3	0.00	31.33	18.67	3.00	1.67	17.33	34.67	4.33
41	PP1505-2-8-1	1.67	38.67	33.00	5.00	2.00	24.33	49.00	5.00
42	PP1505-20-5-2	1.33	23.33	29.00	2.67	0.67	13.00	39.00	8.67
43	PP1505-28-6-1	1.33	31.33	8.33	2.33	1.00	16.67	36.67	3.33

44	PP1505-34-3-6	0.33	31.67	18.00	2.33	2.00	12.00	46.67	6.00
45	PP1505-36-4-1	1.00	29.00	37.67	1.67	1.00	16.33	58.33	7.33
46	PP1505-36-4-2	0.33	20.67	23.00	2.33	0.33	11.67	44.33	7.33
47	PP1505-63-2-4	0.00	23.00	39.33	3.67	0.33	6.00	57.00	11.67
	Fp	0.897	0.340	0.513	0.904	0.798	0.790	0.175	0.608
	Vr	0.71	1.10	0.98	0.71	0.80	0.80	1.26	0.93
	Mean	1.01	29.60	27.50	3.21	1.56	16.78	48.80	6.34
	SE ±	0.87	8.47	9.51	1.35	1.26	5.45	9.01	2.06
	LSD ($P \leq 0.05$)	2.45	23.80	26.71	3.78	3.54	15.29	25.31	5.78
	CV (%)	150.20	49.50	59.90	72.50	139.70	56.20	32.00	56.20

Note: Fp, *F* probability; Vr, variance; SE, standard error of the mean; LSD, least significant difference; CV, coefficient of variation
